# Comparative Transcriptome Analysis Reveals Sex-Biased Gene Expression in Juvenile Chinese Mitten Crab *Eriocheir sinensis*

**DOI:** 10.1371/journal.pone.0133068

**Published:** 2015-07-20

**Authors:** Yuan Liu, Min Hui, Zhaoxia Cui, Danli Luo, Chengwen Song, Yingdong Li, Lei Liu

**Affiliations:** 1 Key Laboratory of Experimental Marine Biology, Institute of Oceanology, Chinese Academy of Sciences, Qingdao, China; 2 National & Local Joint Engineering Laboratory of Ecological Mariculture, Qingdao, China; 3 University of Chinese Academy of Sciences, Beijing, China; Zhejiang University, CHINA

## Abstract

Sex-biased genes are considered to account for most of phenotypic differences between males and females. In order to explore the sex-biased gene expression in crab, we performed the whole-body transcriptome analysis in male and female juveniles of the Chinese mitten crab *Eriocheir sinensis* using next-generation sequencing technology. Of the 23,349 annotated unigenes, 148 were identified as sex-related genes. A total of 29 candidate genes involved in primary sex determination pathways were detected, indicating the sex determination cascade of the mitten crab might be more complex than previously supposed. Differential expression analysis showed 448 differentially expressed genes (DEGs) between the two transcriptomes. Most of DEGs were involved in processes such as metabolism and immunity, and not associated with obvious sexual function. The pathway predominantly enriched for DEGs were related to lysosome, which might reflect the differences in metabolism between males and females. Of the immune DGEs, 18 up-regulated genes in females were humoral immune factors, and eight up-regulated genes in males were pattern recognition receptors, suggesting sex differences of immune defense might exist in the mitten crab. In addition, two reproduction-related genes, *vitellogenin* and *insulin-like androgenic gland factor*, were identified to express in both sexes but with significantly higher level in males. Our research provides the first whole-body RNA sequencing of sex-specific transcriptomes for juvenile *E*. *sinensis* and will facilitate further studies on molecular mechanisms of crab sexual dimorphism.

## Introduction

Sexual dimorphism, which differentiates males and females in morphological, physiological and behavioral characteristics, is a common phenomenon in the animal kingdom. Based on the nearly identical genomes, the phenotypic differences between the sexes are thought to largely result from sex differences in gene expression [1,2]. So far, sex-biased gene expression has been well explored in mammals, birds, fish and insects, particularly in classic model organisms [3–6], however, little attention has been received to such studies in crustaceans.

A number of crustacean species exhibit significant sex differences in their biology and economic value [7]. For example, male crabs usually show higher growth rate and larger size than females when they reach maturation, while female crabs are of higher economic values than males [8,9]. Among the cultured crabs, the Chinese mitten crab *Eriocheir sinensis* (Henri Milne Edwards, 1854) is the most prevalent and commercially important species in China [10]. *E*. *sinensis* is a catadromous crustacean that spends most of its life in freshwater and migrates seawards to breed [11]. Due to the complex life cycle, mitten crabs might have unique regulatory mechanisms involved in crustacean reproduction or sexual development [12,13]. *E*. *sinensis* can serve as a model species of crustaceans to study sexual dimorphism and discover sex-biased gene expression.

Next-generation high-throughput sequencing technologies, such as Illumina/Solexa platform and Ion Proton system, have been used to generate large amounts of transcript sequences and gene expression data for non-model species without sequenced genomes [14,15]. Most studies of sex-biased transcriptome have focused on adult gonads in crustacean, and some sex-related genes have been identified, for example, *Tra-2* [16] in the giant tiger shrimp *Penaeus monodon*, *Dmrt*, *Fem-1* and *vasa* in the green mud crab *Scylla paramamosain* [17], and *Dmc1* and DEAD box family genes in *E*. *sinensis* [12,18,19]. Currently, several studies have reported significant sex-biased gene expression in juvenile stages of fish and insects [20–22]. However, the sex-biased genes by comparing expression before reproductive maturity in crustaceans remain largely unexplored.

Here, we analyzed the whole-body transcriptomes of juvenile *E*. *sinensis* using Ion Proton sequencing technology. This study was designed to enrich the genetic resources for *E*. *sinensis*, to identify new candidate genes involved in sex determination and differentiation, and to detail the different expression patterns between the sexes. Furthermore, differentially expressed genes (DEGs) involved in metabolism, immunity and reproduction were determined and analyzed. Our results are important resources for future research on molecular mechanisms underlying the sexual dimorphism in *E*. *sinensis* and other crustaceans.

## Materials and Methods

### Ethics Statement

The Chinese mitten crabs were captured from Liao River in Panjin, China and no specific permission was required for the sampling area and species because of scientific research purpose. The sampling location is not privately owned or protected. No endangered or protected species were involved in the field sampling. The experiments were performed in accordance with the guidelines on the care and use of animals for scientific purpose set by the Institutional Animal Care and Use Committee (IACUC) of the Chinese Academy of Sciences (No. 2011-2). This study was specifically approved by the Committee on the Ethics of Animal Experiments of the Institute of Oceanology at the Chinese Academy of Sciences. All efforts were made to minimize the suffering of the animals.

### Sample preparation and RNA isolation

Healthy mitten crabs were obtained from a local crab farm in Panjin, China. Five males (1.6 ± 0.4 g) and females (1.9 ± 0.2 g) at the third juvenile instar were pooled, as this stage is the earlier stage to distinguish their sex from morphology [23]. Total RNA was extract from the whole bodies using Trizol reagent (Invitrogen, USA) according to the manufacturer’s protocol. The quality and integrality were determined by Agilent2100 (Agilent, USA). The mRNA was purified from total RNA using Dynabeads mRNA Direct Micro kit (Ambion, USA). The final concentration was determined using a NanoDrop2000 spectrophotometer (Thermo Scientific, USA).

### cDNA library construction and sequencing

Two libraries for male and female juveniles of *E*. *sinensis* were constructed in this study. For library preparation, equal amounts of purified mRNA samples from two biological replicates were pooled together for the cDNA synthesis. The cDNA libraries were prepared using an Ion Total RNA-seq kit v2 (Life Technologies, USA), with 100 ng fragmented mRNA. Adapter ligation, size selection, nick repair and amplification (12 cycles) were performed as described in the Ion Proton protocol associated with the kit. The resulting cDNA libraries were purified by AMPure beads (Beckman Coulter, USA), and their concentrations and sizes were determined by Agilent2100 (Agilent, USA). Emulsion PCR and enrichment steps were performed using an Ion PI Template OT2 200 kit v3 (Life Technologies) according to the manufacturer’s instructions. PI Chips were loaded according to the spinning protocol and sequencing was performed on Ion Proton Sequencer using the Proton 200 sequencing kit (Life Technologies). Base calls were collected with Torrent Suite using v4.0.2 software.

### Sequence assembly

The raw data for each pool of samples were separately trimmed to remove adaptors and low quality regions (< Q20). Reads with a length of less than 50 bp were also discarded. The remaining reads without ambiguous bases (N) were *de novo* assembled in a unique file by Trinity (http://trinityrnaseq.sourceforge.net/) with k-mer length of 25 referring to the strategy of Grabherr et al. [24]. Trinity combining three independent software modules: Inchworm, Chrysalis and Butterfly, applied sequentially to process large volumes of RNA-seq reads into contigs, de Bruijn graphs and full-length transcripts.

### Gene annotation

Gene functional annotation was performed by sequence comparison with public databases. All assembled transcripts were searched against the NCBI non-redundant (nr) protein database (http://www.ncbi.nlm.nih.gov/) using BlastX algorithm with an *E*-value cutoff of 1E-05. The unigenes were obtained after clustering the top hit results. Gene Ontology (GO) annotations were determined using Blast2GO to obtain a functional classification of the unigenes [25]. EggNOG (evolutionary genealogy of genes: Non-supervised Orthologous Groups) was performed to predict and classify potential functions of the unigenes based on known orthologous gene products [26]. EC (Enzyme Commission) terms and biochemical pathway information were generated by Kyoto Encyclopedia of Genes and Genomes (KEGG) database [27]. In addition, the unigenes associated with sex determination and differentiation were identified manually according to the annotation in consulting published literature and public datasets.

### Identification of SNPs and SSRs

Single nucleotide polymorphisms (SNPs) were identified using SAMtools mpileup and VarScan (version 2.3.3) [28,29]. VarScan was run with the following parameters: “mpileup—p-value 0.01—min-avg-qual 20—min-reads2 2—min-var-freq 0.2-variants”. From these results, a set of high confidence SNPs (with coverage of 8 or more reads) were identified. All unigenes were searched for the presence of SSRs using MISA (http://pgrc.ipk-gatersleben.de/misa/) [30] with the following minimum length criteria (unit size/minimum repeat time): 2/6, 3/5, 4/5, 5/5 and 6/5. Compound microsatellites were defined as repeats interrupted by a non-repetitive sequence of a maximum of 100 nucleotides.

### Differential expression analysis

The reads for a specific transcript were counted by mapping reads to assembled unigene sequences. The unigene expression was calculated using the reads per kb per million reads (RPKM) method [31]. Differentially expressed genes (DEGs) were identified by the DESeq program [32]. The fold change values > 2 and false discovery rates (FDR) adjusted significance values < 0.05 (-log10 (0.05) = 1.3) were considered as the threshold to judge the significance of unigene expression.

GO, eggNOG, KEGG Orthology (KO) and KEGG pathway enrichment analyses were used to categorize DEGs and evaluate DEGs in the potential biological pathways. Processes, functions or components in the GO and KEGG pathway enrichment analyses with *p*-values less than 0.05 (-log10 (0.05) = 1.3) were considered to be significantly different in the DEGs. Based on public databases and the published literatures, the crucial DEGs related to metabolism, immunity and reproduction were manually checked.

The DEG encoding insulin-like androgenic gland factor (*IAG*) was selected for further sequence and phylogenetic analysis. Multiple amino acid sequence alignment was performed using the Clustal X with the default settings [33]. Neighbor-joining tree with bootstrap values were constructed for phylogenetic analysis using MEGA 4.0 [34]. All the reference sequences for phylogenetic analysis were derived from GenBank.

### Quantitative Real-time PCR (qRT-PCR) validation

To validate RNA-seq data and expression profiles obtained from DESeq analysis, mitten crabs at the same developmental stage, the independent samples of RNA-seq, were used for real-time PCR analyses. Three biological replicates were prepared for male and female juveniles. Approximately 2.5 μg of total RNA of each sample obtained as previously described were treated with DNase I (Ambion, USA) at 37°C for 1 h, reverse-transcribed using M-MLV reverse transcriptase (Promega, China) and amplified by qRT-PCR.

The SYBR Green I RT-PCR assay was carried out in an ABI PRISM 7300 Sequence Detection System (Applied Biosystems, USA). The gene-specific primers designed for the 12 genes are listed in S1 Table. These 12 genes all exhibited large significant differences in expression between male and female libraries. The *β-actin* from *E*. *sinensis* was chosen as reference gene for internal standardization. The PCR was carried out in a total volume of 10 μl, containing 5 μl of 2×SYBR Premix Ex Taq (TaKaRa, China), 0.2 μl 50×ROX Reference Dye, 2 μl of the diluted cDNA mix, 0.2 μl of each primer (10 μM), and 2.4 μl of sterile distilled H_2_O. The PCR program was 95°C for 5 min, followed by 40 cycles of 95°C for 5 s and 60°C for 31 s. Each sample was run in triplicate along with the internal control gene. To confirm that only one PCR product was amplified and detected, dissociation curve analysis of amplification products was performed at the end of each PCR reaction. After the PCR program, data were analyzed with ABI7300 SDS software (Applied Biosystems). Fold change for the gene expression relative to controls was determined by the 2^-ΔΔCt^ method [35]. All data were given in terms of relative mRNA expression as mean ±S.E. The results were subjected to one-way analysis of variance (one way ANOVA) using SPSS 13.0, and the *P* values less than 0.05 and 0.01 were considered statistically significant.

## Results

### Transcriptome sequencing and assembly

Two cDNA libraries were generated with pooled mRNAs from the whole bodies of female and male juveniles of *E*. *sinensis*. After quality trimming and the removal of adapters, sequencing runs performed on Ion Proton platform produced a total of 42,979,050 reads for the female and 47,560,370 reads for the male. All data were deposited in NCBI Short Read Archive database with accession numbers SRX554564 and SR554562. Clustering and assembly of these reads resulted in 282,954 contigs with an average length of 274 bp. Further assembly analysis showed all contigs contributed to 151,128 transcripts with an average length of 614 bp (S2 Table).

### Functional annotation

BlastX searches of all assembled transcripts against the NCBI nr database revealed 23,349 unigenes with significant matches to existing protein sequences above the preset cutoff value. The average length of these annotated unigenes was 988 bp with N50 length of 1,375 bp (S2 Table).

Based on GO analysis, 17,388 unigenes (74.5%) were assigned to one or more GO term. Finally, 77,697 GO assignments were obtained and fall into the three major functional categories (S1 Fig). Thereinto, 38,578 unigens (49.7%) were involved in biological process, 26,583 (34.2%) were cellular components and 12,518 (16.1%) have molecular functions. Moreover, 0.4% (323 unigenes) were assigned to reproduction of biological process.

To classify orthologous gene products, 22,101 unigenes were assigned to 25 function categories (S2 Fig). Of these, the cluster of ‘function unknown’ (4,825, 18.3%) represented the largest group, followed by ‘signal transduction mechanisms’ (3,757, 14.3%), ‘general function prediction only’ (3194, 12.1%) and ‘transcription’ (2,112, 8.0%). KEGG analysis revealed that 9,605 unigenes were assigned to six biochemical pathways, including metabolism, genetic information processing, environmental information processing, cellular processes, organismal systems and human diseases. A total of 3,743 unigenes had significant matches in the KEGG database with corresponding EC numbers (S2 Table).

### SNP and SSR discovery

**Fig 1:**
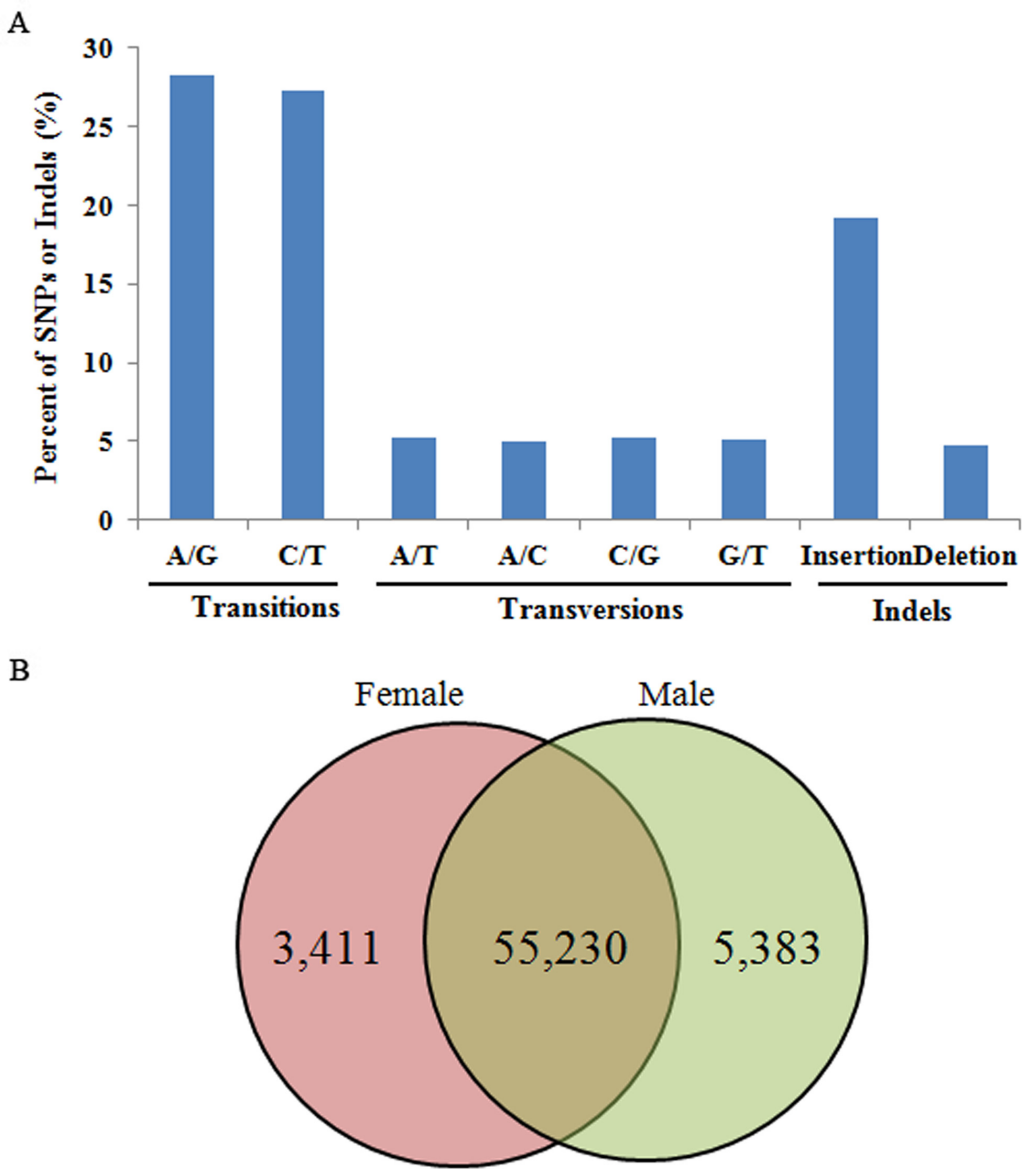
Summary statistics of single nucleotide polymorphisms (SNPs) including indels from female and male *Eriocheir sinensis* unigenes. (A) Classification of SNPs. Distribution (%) of each SNP and indel type. (B) Venn diagram of common and unique SNPs and indels of female and male unigenes.

Using SAMtools/VarScan software, 48,753 SNPs and 15,271 indels were identified from *E*. *sinensis* unigenes. The overall frequency of all types of SNPs, including indels, was one per 360 bp (Fig 1A). Transition occurred 2.7 times more frequently than transversion. A/G was the most abundant transition (28.3%), and A/T was the most abundant transversion (5.3%). Indels were less frequent than transitions, with a frequency of one per 1,511 bp and a total proportion of around 23.9%. Of the identified SNPs and indels, 55,230 were found in both female and male *E*. *sinensis* unigenes. 3,411 SNPs and indels were found exclusively in female unigenes, while 5,383 were just present in male unigenes (Fig 1B).

In addition, using MISA, 3,928 perfect and 262 compound SSRs were detected from *E*. *sinensis* unigenes (S3 Table). Trinucleotide repeats were the most frequent type, counting a total number of 2,286 (54.6%) with AGG/CCT as a major motif accounted for 31.4% of all trinucleotide repeats. Dinucleotides repeats were the second most frequent type accounting for 37.3% of all SSRs with the abundance of AC/GT motif. And the other two distinguished types, compound and tetranucleotide repeats, accounted for less than 10% of all SSRs.

### Genes related to sex determination and differentiation

Of the total unigenes, 148 were identified as sex-related genes based on sequence annotation and the published literature (S4 Table). These genes could be categorized into 36 groups and putatively involved in sex determination and differentiation, oogenesis, spermatogenesis and gonad development. Among these unigenes, 40 were classified as testis or sperm-specific genes, and 24 were ovary or oocyte-specific genes. Eleven unigenes annotated as *SOX* (SRY related HMG box) gene family were found, including *SOX2*, *SOX5*, *SOX8*, *SOX14*, *SOXB1*, *SRY*, *SRY-box containing protein 8* and *B2b2*.

A total of 29 genes with similarity to those involved in primary sex determination pathway were identified (Table 1). Eight genes, including two fruitless (*Fru*), two transformer-2 (*Tra-2*) and four feminization-1 (*Fem-1*) genes, were associated with the sex determination in *Drosophila melanogaster* and *Caenorhabditis elegans*. Fourteen genes involved in mammalian female sexual development were detected: five genes belonging to the WNT signaling pathway (*RSPO-1*, *WNT4* and *β-catenin*), a transcription factor (*FOXL2*), three estradiol receptors (*ER*), and five activin-binding proteins (*FST*). Seven genes involved in mammalian male sexual development were examined: a doublesex and mab-3 related transcription factor 2 (*Dmrt2*), three prostaglandin D synthases (*PGDS*), and three genes belonging SOX transcription factors (*SRY* and *SOX8*).

**Table 1:** Primary sex determination pathway related genes detected in *Eriocheir sinensis* transcriptomes.

Gene	Unigene	Isoform name	Gene annotation [Matched species]	*E*-value
**Genes involved in *Drosophila melanogaster* sex determination pathway**				
*Fru*	comp31866_c0_seq1	EsFru	fruitless [*Chorthippus biguttulus*]	1.95863E-10
	comp21702_c0_seq1	EsFru-like	sex determination protein fruitless-like [*Ceratitis capitata*]	3.21181E-23
*Tra-2*	comp74937_c1_seq33	EsTra-2	transformer-2 protein [*Penaeus monodon*]	1.6998E-46
	comp74937_c1_seq2	EsTra-2v	variant transformer-2 protein [*Penaeus monodon*]	2.18253E-45
**Genes involved in *Caenorhabditis elegans* sex determination pathway**				
*Tra-2*	comp74937_c1_seq33	EsTra-2	transformer-2 protein [*Penaeus monodon*]	1.6998E-46
	comp74937_c1_seq2	EsTra-2v	variant transformer-2 protein [*Penaeus monodon*]	2.18253E-45
*Fem-1*	comp73673_c0_seq4	EsFem-1	fem-1-like protein [*Daphnia pulex*]	4.22441E-59
	comp62108_c0_seq1	EsFem-1B	fem-1 homolog B-like protein [*Locusta migratoria manilensis*]	0
	comp73673_c0_seq1	EsFem-1C	fem-1-like protein C [*Crassostrea gigas*]	1.1536E-175
	comp69377_c0_seq1	EsFem-1hC	fem-1 homolog C-like protein [*Locusta migratoria manilensis*]	7.84219E-12
**Genes involved in mammalian sex determination and sexual differentiation pathway**				
Ovarian development				
*β-catenin*	comp76922_c0_seq8		beta-catenin [*Parhyale hawaiensis*]	4.7035E-113
*ER*	comp66193_c2_seq1	EsER1	estradiol receptor-like protein 1 [*Portunus trituberculatus*]	7.15127E-48
	comp55365_c1_seq1	EsER2	estradiol receptor-like protein 2 [*Portunus trituberculatus*]	3.25746E-17
	comp68632_c0_seq1	EsER3	estradiol receptor-like protein 3 [*Portunus trituberculatus*]	0
*FOXL2*	comp56545_c0_seq1		forkhead box protein L2 [*Astyanax mexicanus*]	3.7583E-26
*FST*	comp48310_c0_seq1	EsFST	Follistatin [*Acromyrmex echinatior*]	1.31862E-34
	comp43834_c0_seq1	EsFSTp	Follistatin precursor [*Pediculus humanus corporis*]	3.11653E-08
	comp89797_c0_seq1	EsFST5	follistatin-like 5 [*Scylla paramamosain*]	8.94175E-30
	comp86047_c0_seq1	EsFST5-like	follistatin-related protein 5-like [*Nasonia vitripennis*]	1.52671E-56
	comp35850_c0_seq1	EsFST5-like2	follistatin-related protein 5-like [*Acyrthosiphon pisum*]	7.3293E-62
*RSPO-1*	comp92691_c0_seq1	EsRSPO-1	R-spondin 1 [*Glandirana rugosa*]	5.20609E-06
	comp26865_c0_seq1	EsRSPO-1p	R-spondin-1 precursor [*Oryzias latipes*]	9.0604E-25
*WNT4*	comp30980_c0_seq1	EsWNT4	protein Wnt-4-like [*Bos taurus*]	1.53802E-56
	comp9493_c0_seq1	EsWNT4s	putative secreted signaling factor WNT4 [*Daphnia pulex*]	5.87015E-09
Testis development				
*Dmrt2*	comp18195_c0_seq1		doublesex and mab-3 related transcription factor 2 [*Xenopus laevis*]	2.46563E-10
*PGDS*	comp58387_c0_seq1	EsPGDS	prostaglandin D synthase [*Eriocheir sinensis*]	1.8541E-125
	comp53351_c0_seq1	EsGPGDS	glutathione-dependent prostaglandin D synthase [*Penaeus monodon*]	1.33867E-56
	comp73984_c0_seq5	EsHPGDS	hematopoietic prostaglandin D synthase [*Penaeus monodon*]	2.16344E-48
*SRY*	comp34840_c0_seq1	EsSRY	Sex-determining region Y protein [*Pediculus humanus corporis*]	3.57723E-46
*SOX8*	comp21432_c0_seq1	EsSRY8	SRY-box containing gene 8 [*Rattus norvegicus*]	6.87426E-11
	comp71497_c0_seq3	EsSOX8	Transcription factor SOX-8 [*Camponotus floridanus*]	3.46753E-28

The primary sex determination pathway related genes were expressed in both female and male *E*. *sinensis* transcriptomes (S3 Fig). EsPGDS was the most highly expressed transcript (RPKM value > 800), followed by EsER3 (RPKM value > 50) and EsHPGDS (RPKM value > 40). EsDmrt2, EsPGDS and EsSRY presented higher expression pattern in males (fold change values > 1.5), while EsFST5, EsRSPO-1p and EsFST5p showed preferential expression in females (fold change values > 1.5). The other 23 genes were similar expressed in females and males.

From the 148 unigenes annotated for sex-related genes, 18 SNPs were identified in female individuals and 23 in male individuals (S5 Table). Most unigenes had only one SNP, except for vitelline membrane outer layer protein I, SOX14, oocyte zinc finger protein, male reproductive-related microfibril-associated protein spermatogenesis-associated protein 5 and 20. In addition, five compound and 18 perfect SSRs were detected from the sex-related unigenes (S6 Table). The size of SSRs ranged from 12 to 242, with trinucleotide repeats as the most frequent type. Three sex-related genes, including SRY-box containing gene 8, SRY interacting protein 1 and zinc finger protein 76, were found to contain both SNPs and SSRs.

### Differential expression between the sexes

The DESeq method identified 448 differentially expressed unigenes between the two transcriptomes, including 188 up-regulated in females and 260 up-regulated in males (S7 Table). The distribution of the significant changes detected was illustrated in a volcano plot, where the statistical significance of each unigene was plotted against fold change (S4 Fig). Sequences with the highest average differences between the sexes also had the smallest false discovery rate (FDR) values.

**Fig 2:**
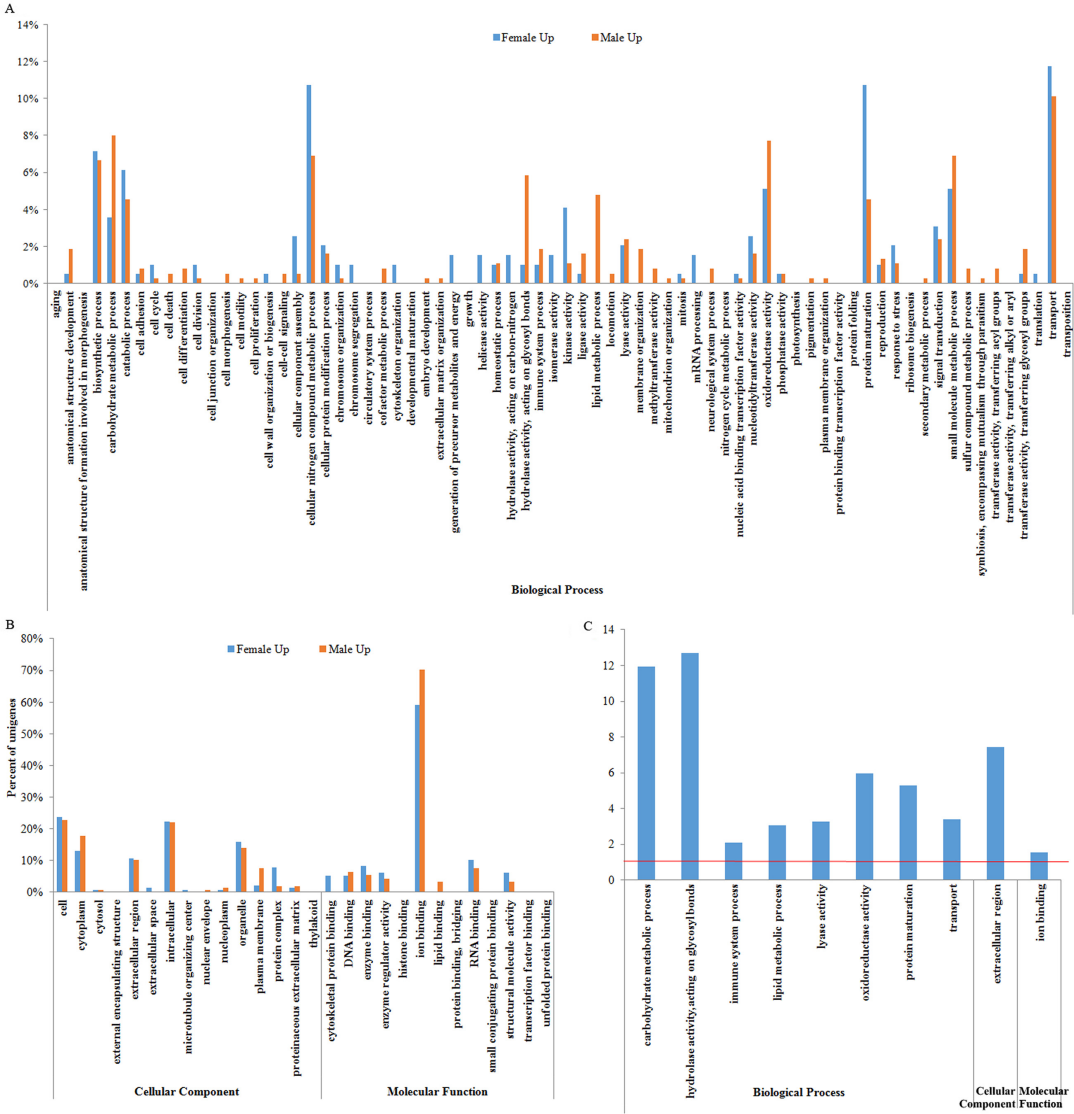
GO distributions of differentially expressed genes (DEGs) from female and male *Eriocheir sinensis* transcriptomes. (A) Different functional distribution of the DEGs involved with biological processes. (B) Different functional distribution of the DEGs involved with cellular components and molecular functions. (C) Differentially expressed functional processes. The horizontal line indicates the significance threshold (*p* < 0.05).

All of the DEGs were performed on GO function and pathway enrichment analysis. Based on GO analysis, a total of 340 DEGs with GO terms were categorized into three major functional groups (Fig 2). Within the biological processes, the highest percentage of DEGs involved in GO terms was ‘transport’ (10.7%), followed by ‘carbohydrate metabolic process’ (8.2%), ‘biosynthetic process’ (6.8%), ‘oxidoreductase activity’ (6.8%), ‘protein maturation’ (6.6%) and ‘carbohydrate metabolic process’ (6.5%) (Fig 2A). Of the cellular component categories, the majority of GO terms were related to ‘cell’ (23.1%), ‘intracellular’ (22.1%) and ‘cytoplasm’ (15.4%) (Fig 2B). As for molecular functions, the three major GO terms were ‘ion binding’ (64.6%), ‘RNA binding’ (8.9%) and ‘enzyme binding’ (6.8%) (Fig 2B). After the overall comparison was completed, the top ten significantly changed categories were obtained, including ‘hydrolase activity, acting on glycosyl bonds’, ‘carbohydrate metabolic process’, ‘extracellular region’, ‘oxidoreductase activity’, ‘protein maturation’, ‘transport’, ‘lyase activity’, ‘lipid metabolic process’, ‘immune system process’ and ‘ion binding’ (*p* < 0.05, Fig 2C).

**Fig 3:**
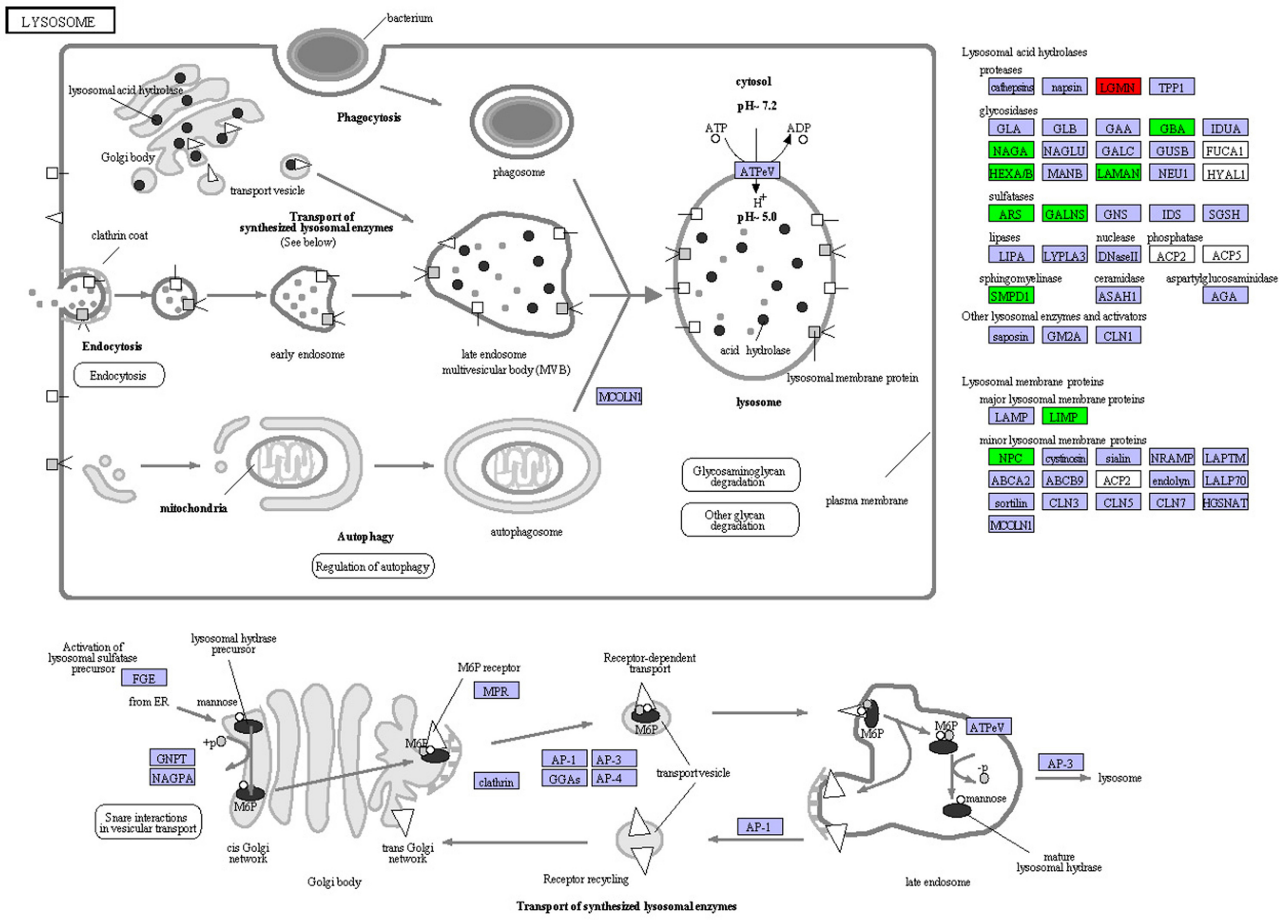
Expression pattern of genes involved in lysosomal pathway. The pathway is based on a KEGG pathway analysis. The up-regulated genes in male and female are labeled by green and red, respectively, and the purple color represents genes with no expression differences between female and male transcriptomes of *Eriocheir sinensis*.

KEGG analysis revealed that 139 DEGs with KO terms were involved in 29 predicated biological pathways (S5 Fig). The most abundant pathways included ‘carbohydrate metabolism’ (36 DGEs), ‘glycan biosynthesis and metabolism’ (24 DGEs), ‘transport and catabolism’ (24 DGEs) and ‘lipid metabolism’ (16 DGEs). The significantly distinct categories were ‘carbohydrate metabolism’, ‘glycan biosynthesis and metabolism’, ‘metabolism of cofactors and vitamins’, ‘transport and catabolism’, ‘digestive system, ‘lipid metabolism’, ‘xenobiotics biodegradation and metabolism’ and ‘amino acid metabolism’ (*p* < 0.05, S5 Fig). Except for metabolic pathway, the pathway predominantly enriched for DEGs were related to lysosome (ko04142) of ‘transport and catabolism’ (Fig 3). There were 19 DEGs with ten KO terms in lysosomal pathway, including 18 up-regulated in males and one up-regulated in females. In addition, one important pathway identified as enriched for the up-regulated genes in males were associated with sphingolipid metabolism (ko00600) of ‘lipid metabolism’ (S6 Fig).

### Candidate DEGs involved in metabolism, immunity and reproduction

By sequence annotation and functional classification, we identified 61 differentially expressed unigenes related to metabolism, immunity and reproduction. The metabolism-related DGEs could be categorized into four classes, including five female-biased genes in amino acid metabolism, ten male-biased genes in lipid metabolism, six male-biased genes in glycan metabolism, and three male-biased genes in cytochrome P450 (CYP450) superfamily (Table 2). A total of 30 immunity-related DGEs were found, including 18 up-regulated humoral immune factors in females, eight up-regulated pattern recognition receptors (PRRs) (*CLEC* and *LGBP*) and four other up-regulated immune factors in males (Table 3). The female-biased immune DGEs were largely involved in antimicrobial peptide synthesis (*Lyz*, *Crustin* and *Carcinin*), prophenoloxidase (proPO) system (*SP*, *PPAF* and *Kazal*) and antioxidant system (*SOD* and *Trx*). Of the reproduction-related DGEs, two genes involved in ovarian development showed female-biased expression, while five genes, including two *Vg* homologs, *VMO1*, *IAG* and *NPC2*, showed male-biased expression (Table 4).

**Table 2:** List of differentially expressed genes related to metabolism in female and male *Eriocheir sinensis* transcriptomes.

Gene	Unigene	Gene annotation [Matched species]	Fold change (Female: Male)
**Up-regulated genes in females**			
Amino acid metabolism-related genes			
*AspRS*	comp59739_c0_seq1	aspartate-tRNA ligase, mitochondrial-like [*Takifugu rubripes*]	3.17
*Ark*	comp72982_c2_seq1	arginine kinase [*Eriocheir sinensis*]	2.32
*Arg1*	comp74968_c0_seq2	arginase-1 isoform 2 [*Trichechus manatus latirostris*]	2.08
*NIT2*	comp56883_c0_seq1	Nit protein 2-like [*Saccoglossus kowalevskii*]	7.86
	comp48414_c0_seq2	Omega-amidase NIT2-B [*Crassostrea gigas*]	6.85
**Up-regulated genes in males**			
Lipid metabolism-related genes			
*ACAC*	comp77110_c0_seq6	Acetyl-CoA carboxylase [*Harpegnathos saltator*]	0.47
*ASAH2*	comp73699_c0_seq4	ceramidase [*Danaus plexippus*]	0.42
*ARSA*	comp74420_c0_seq3	arylsulfatase A-like [*Strongylocentrotus purpuratus*]	0.29
*FASN*	comp75785_c0_seq2	fatty acid synthase [*Tribolium castaneum*]	0.43
	comp76291_c0_seq1	fatty acid synthase-like isoform 1 [*Nasonia vitripennis*]	0.46
*GBA*	comp74162_c1_seq1	glucosylceramidase-like [*Ciona intestinalis*]	0.46
	comp63076_c0_seq1	glucosylceramidase-like [*Monodelphis domestica*]	0.47
*Se-Gpx*	comp66076_c0_seq2	selenium-dependent glutathione peroxidase [*Procambarus clarkii*]	0.21
*SMPD1*	comp72185_c1_seq1	acid sphingomyelinase 1 [*Glossina morsitans morsitans*]	0.36
*PLRP2*	comp75117_c0_seq1	pancreatic lipase-related protein 2-like [*Acyrthosiphon pisum*]	0.34
Glycan metabolism-related genes			
*A4GALT*	comp9581_c0_seq1	lactosylceramide 4-alpha-galactosyltransferase [*Rattus norvegicus*]	0.33
*GALNS*	comp76195_c0_seq5	n-acetylgalactosamine-6-sulfatase-like [*Monodelphis domestica*]	0.40
	comp76195_c0_seq6	galactosamine (N-acetyl)-6-sulfate sulfatase [*Xenopus (Silurana) tropicalis*]	0.49
*DHDH*	comp63990_c0_seq2	Trans-1,2-dihydrobenzene-1,2-diol dehydrogenase [*Acromyrmex echinatior*]	0.44
*HEXA_B*	comp75397_c0_seq1	beta-hexosaminidase subunit alpha-like [*Ornithorhynchus anatinus*]	0.43
*LAMAN*	comp60626_c0_seq1	lysosomal alpha-mannosidase precursor [*Danio rerio*]	0.40
Cytochrome P450 superfamily			
*CYP3A4*	comp59688_c0_seq1	cytochrome P450, family 3, subfamily A, polypeptide 4 [*Callorhinchus milii*]	0.08
*CYP9Z7*	comp75041_c0_seq1	cytochrome P450 9Z7 [*Tribolium castaneum*]	0.16
*CYP2B19*	comp69705_c0_seq2	cytochrome P450 2B19-like [*Ciona intestinalis*]	0.48

**Table 3:** List of differentially expressed genes related to immunity in female and male *Eriocheir sinensis* transcriptomes.

Gene	Unigene	Gene annotation [Matched species]	Fold change (Female: Male)
**Up-regulated genes in females**			
*Lyz*	comp67931_c0_seq2	lysozyme [*Scylla paramamosain*]	7.21
*A2ML2*	comp75726_c0_seq17	alpha2 macroglobulin isoform 2 [*Fenneropenaeus chinensis*]	2.15
*Carcinin*	comp61957_c0_seq1	carcinin-like protein [*Carcinus maenas*]	2.72
*Crustin*	comp54631_c0_seq1	crustin [*Scylla tranquebarica*]	3.94
	comp66850_c0_seq1	crustin 2 [*Portunus trituberculatus*]	2.06
*CPAMD8*	comp74090_c0_seq4	C3 and PZP-like alpha-2-macroglobulin domain-containing protein 8-like [*Metaseiulus occidentalis*]	2.07
*HPT factor 9*	comp58745_c0_seq1	HPT factor 9 [*Pacifastacus leniusculus*]	2.25
*Kazal*	comp67267_c0_seq3	Kazal-type protease inhibitor [*Eriocheir sinensis*]	2.25
*PPAF*	comp72920_c0_seq1	prophenoloxidase activating factor serine proteinase [*Scylla serrata*]	7.33
	comp57767_c0_seq1	phenoloxidase activating factor [*Portunus trituberculatus*]	2.32
*SP*	comp67291_c1_seq3	clip domain serine proteinase 3 [*Portunus trituberculatus*]	2.63
	comp71922_c0_seq2	clip domain serine proteinase [*Portunus trituberculatus*]	2.30
	comp63300_c1_seq1	clip domain serine protease [*Eriocheir sinensis*]	2.11
	comp67168_c0_seq1	serine proteinase [*Portunus trituberculatus*]	2.06
	comp67235_c0_seq1	trypsin-like serine protease [*Eriocheir sinensis*]	2.16
*SOD*	comp66967_c0_seq1	superoxidase dismutase [*Eisenia fetida*]	2.28
*PDGF/VEGF-related factor 1*	comp75589_c0_seq4	PDGF/VEGF-related factor 1 [*Eriocheir sinensis*]	2.26
*Trx1*	comp61426_c0_seq2	Trx1 [*Eriocheir sinensis*]	2.08
**Up-regulated genes in males**			
*CLEC*	comp62386_c0_seq1	C-type lectin [*Eriocheir sinensis*]	0.29
	comp74386_c0_seq1	C-type lectin [*Eriocheir sinensis*]	0.37
	comp69162_c2_seq4	C-type lectin 1 [*Marsupenaeus japonicus*]	0.34
	comp18326_c0_seq1	C-type lectin-1 [*Litopenaeus vannamei*]	0.45
	comp22144_c0_seq1	C-type lectin-2 [*Litopenaeus vannamei*]	0.37
*CLR*	comp45203_c0_seq1	C-type lectin receptor protein [*Eriocheir sinensis*]	0.46
*HC6*	comp67750_c0_seq2	hemocyanin subunit 6 [*Eriocheir sinensis*]	0.50
*LGBP*	comp56987_c0_seq1	lipopolysaccharide and beta-1,3-glucan binding protein [*Procambarus clarkii*]	0.19
	comp72288_c0_seq1	lipopolysaccharide and beta-1,3-glucan binding protein [*Eriocheir sinensis*]	0.29
*MIF*	comp54328_c1_seq1	macrophage migration inhibitory factor [*Anisakis simplex*]	0.40
*Peritrophin*	comp54270_c0_seq1	peritrophin [*Macrobrachium nipponense*]	0.39
*PLGRKT*	comp59036_c0_seq2	plasminogen receptor (KT) [*Dasypus novemcinctus*]	0.40

**Table 4:** List of differentially expressed genes related to reproduction in female and male *Eriocheir sinensis* transcriptomes.

Gene	Unigene	Gene annotation [Matched species]	Fold change (Female: Male)
**Up-regulated genes in females**			
*Vg receptor*	comp76488_c0_seq9	vitellogenin receptor [*Macrobrachium rosenbergii*]	2.18
*Pxt*	comp58063_c0_seq3	chorion peroxidase-like [*Acyrthosiphon pisum*]	2.16
**Up-regulated genes in males**			
*Vg*	comp10184_c0_seq1	vitellogenin [*Crassostrea gigas*]	0.19
	comp77225_c0_seq2	vitellogenin [*Charybdis feriata*]	0.47
*VMO1*	comp67371_c0_seq1	vitelline membrane outer layer protein 1 homolog isoform 1 [*Oryzias latipes*]	0.38
*IAG*	comp61312_c0_seq3	insulin-like androgenic gland factor [*Callinectes sapidus*]	0.25
*NPC2*	comp55602_c0_seq1	epididymal secretory protein E1-like [*Hydra magnipapillata*]	0.36

A partial sequence of 741 bp (comp61312_c0_seq3), without an ATG codon but with a TAA stop codon at position 526 bp, was identified to encode an IAG ortholog (EsIAG, S7 Fig). The deduced amino acid sequence was 132 aa in length and contained B chain and A chain, with three disulfide bridges (between C_B9_ and C_A12_, C_B20_ and C_A25_, C_A11_ and C_A16_, respectively). Multiple sequence alignment of EsIAG and six decapod IAGs showed the conserved cysteine residues shared by all sequences (S7A Fig). EsIAG displayed 36.3% amino acid identity with *Callinectes sapidus* IAG1, 32.5% with *C*. *sapidus* IAG2 and 33.1% with *Scylla paramamosain* IAG. The phylogenetic tree showed that the IAGs formed two major clades: one with three isopods and the other containing five subclades from the decapods (S7B Fig). The crab IAGs were separated in two gruops. EsIAG was clustered with IAGs from the Atlantic blue crab *C*. *sapidus* and the mud crab *S*. *paramamosain* to form one group. Another group included IAG from the blue swimmer crab *Portunus pelagicus*, which had a closer relationship with two IAGs from crayfish.

### qRT-PCR validation of RNA-seq data

**Fig 4:**
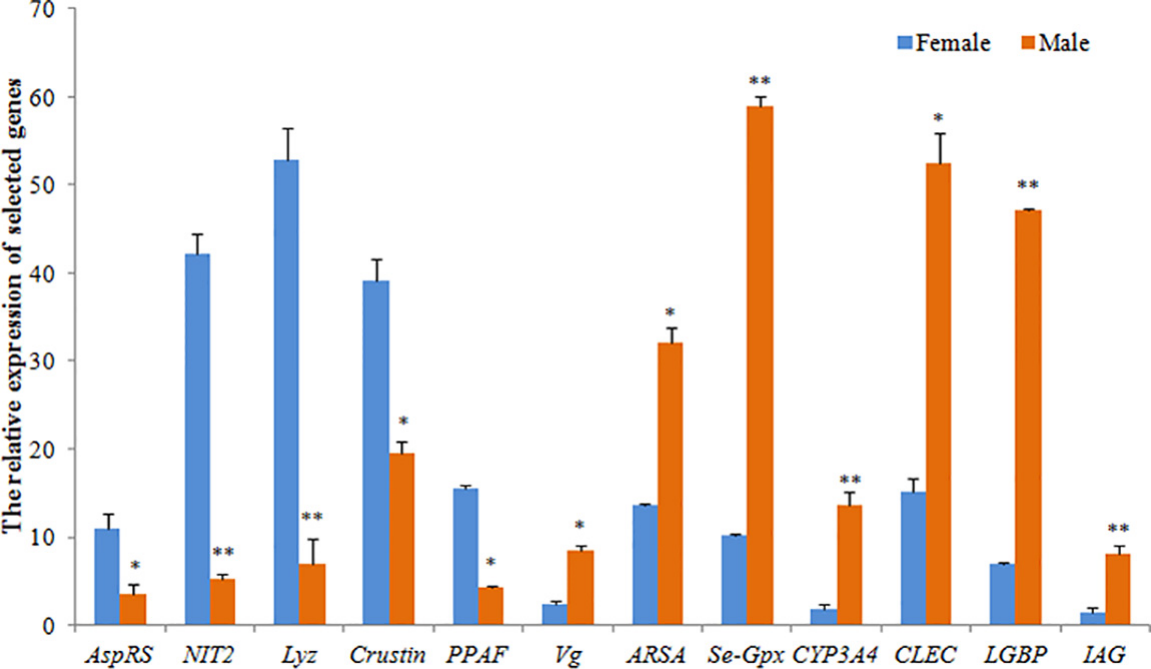
qRT-PCR validation of selected differentially expressed genes identified by RNA-seq. Vertical bars represent the mean ± S.E. (n = 3). Significant differences are indicated with an asterisk at *P* < 0.05, and two asterisks at *P* < 0.01.

To verify the result of RNA-seq analysis, 12 DGEs based on RNA-seq were selected for qRT-PCR to further investigate the expression profiles. The expression patterns from qRT-PCR showed general agreement with those from the RNA-seq (Fig 4). Five genes including *AspRS*, *NIT2*, *Lyz*, *Crustin* and *PPAF* were up-regulated in females, while *Vg*, *ARSA*, *Se-Gpx*, *CYP3A4*, *CLEC*, *LGBP* and *IAG* were up-regulated in males. Among these genes, *NIT2* showed the largest up-regulation in females and CYP3A4 manifested the largest up-regulation in males, which was consistent with RNA-seq results. The consistent expression between qRT-PCR and RNA-seq analyses confirmed the accuracy of Proton results.

## Discussion

### Whole-body reference transcriptome of male and female *Eriocheir sinensis*

Compared with the available data from database and the previously reported transcriptomes from male gonads, the present study, providing more than 10 Gb clean data, is the first whole-body RNA sequencing of sex-specific transcriptomes for juvenile Chinese mitten crab *E*. *sinensis*. This resource expands the limited amount of sex-related sequence data of *E*. *sinensis*, which will facilitate further molecular investigation on sexual dimorphism of crab.

Similar to recent studies in *E*. *sinensis* [36–38], only about 15% of all assembled transcripts are successfully matched in the nr database, probably due to the limited number of crustacean sequences in public database. However, this percentage is higher than that in gonad transcriptomes of *S*. *paramamosain* [17], supposedly because the whole bodies of juvenile are used and more extensive sequencing depth are applied in our transcriptomes. From our sequencing effort, a large number of SNPs and SSRs are detected for future genetics studies. This database, especially the sex-related markers, will play important roles in the exploration and utilization of sex-related genes, and may provide powerful tools for early gender identification and breeding in this commercially important species.

### Candidate genes involved in sex determination and differentiation

Though several sex-related genes were obtained previously [39–41], little information about sex determination cascades is available in the mitten crab. GO analysis and orthology prediction supply gene function classification labels and an overall framework for the sex-specific transcriptomes.

Two *Tra-2* and two *Fru* genes are identified in our transcriptome database. The *Tra-2* and *Fru* genes, by promoting female sexual development or directing male sexual behavior, have been proved to participate in the *Drosophila* sex determination cascade [42,43]. Together with the previously cloned *sex-lethal* (S*xl*) [41] and our identified *double sex* (*dsx*) [44], most of ortholog genes in ‘Sxl-Tra/Tra-2-Dsx/Fru’ pathway are detected in the mitten crab. As suggested in the penaeid shrimp [16,45], the present data provides a hint that the mitten crab might adopt a similar sex determination pathway with that in *Drosophila*. However, the absence of *Tra* and lack of a sex specificity of *Sxl* in the mitten crab, also reported in the silkworm *Bombyx mori* [46–48], imply that genetic regulation for sex determination potentially does not initiate from *Sxl*.

We discover four homologs of *Fem-1* that have not been identified previously in *E*. *sinensis*. In *C*. *elegans*, *Fem-1*, encoding an ankyrin repeat protein Fem-1, is a component of the signal transduction pathway controlling sex determination [49]. In our high density linkage map of *E*. *sinensis*, another ankyrin repeat-containing gene is found to be located on the putative sex chromosome, suggesting its possible role in sex determination [44]. However, in the present study, the four homologs of *Fem-1* are expressed similarly in both males and females. Whether *Fem-1* participates in the process of sex differentiation in the crab has yet to be established.

Besides the above genes, many genes involved in mammalian sex determination cascade are reported in *E*. *sinensis*, further indicating the complex sex determination system of the mitten crab. In mammals, the presence of the male-determining *SRY* gene directs the undifferentiated gonad to develop into testes by promoting the expression of *SOX9* [50]. Early ovarian development has long been considered to be a default pathway switched on passively by the absence of *SRY* gene. However, recent reports have revealed that *FOXL2*-leading pathway and *RSPO-1*-activating signaling pathway act independently and complementary to each other to promote ovarian development [51–53]. In invertebrates, orthologs of *FOXL2* have been characterized, but without a good understanding of their role in reproduction [54–57]. Though the orthologs genes in ‘RSPO-1/WNT4/β-catenin’ signaling pathway are detected, further investigations are needed to determine whether this pathway exists in the mitten crab.

### Patterns of gene expression between the sexes

Differential expression analysis reveals 188 and 260 significantly expressed unigenes in female and male transcriptomes, respectively. These results show a slight imbalance in favor of the male sequences. This tendency has also been reported in adult transcriptomes of *Caligus rogercresseyi* [58] and gonad transcriptomes of the green mud crab [17] and other species such as *Acipenser fulvescens* [59] and *Haliotis rufescens* [60]. Coinciding with that in male and female rainbow trout embryos [20], most DEGs in this study are not related to sexual function. Further GO and KEGG analyses reveal these DEGs are largely involved in biological processes, such as lipid metabolism, glycan metabolism, transport and catabolism, and immune system process. This suggests that there are inherent and broad differences in the transcriptomes of male and female mitten crab, and that these differences are present before sexual maturation.

Lysosomes are membrane-enclosed organelles that contain an array of enzymes capable of breaking down all types of biological polymers-proteins, nucleic acids, carbohydrates and lipids [61]. Of DEGs involved in lysosomal pathway, one gene encoding protease legumain is up-regulated in females, while other genes encoding glycosidases, sulfatases, sphingomyelinase and membrane proteins are up-regulated in males. Together with the identified DEGs related to metabolism, this differential expression pattern might due to the higher level of amino acid metabolism in females as well as higher levels of glycan and lipid metabolisms in males. Three metabolism-related *CYP450* genes, *CYP2B19*, *CYP3A4* and *CYP9Z7*, were identified as male-biased DGEs. Enzymes in CYP2 and CYP3 families, especially *CYP3A4*, have important roles in steroid biosynthesis and metabolism in human [62]. The higher transcripts of CYP450 enzymes in males might indicate male mitten crabs require a large amount of steroid during early juvenile stages.

Sex differences in the immune defense, where females show greater immunity or resistance to infection, have been demonstrated for several arthropods [63–67]. In many cases, females have higher levels of hemocytes or PO activity than males in the absence of infection. Most of these studies have focused on insects such as butterflies, crickets, dragonflies and scorpionflies, with relatively few studies on crustaceans. Here, we first report some genes that potentially contribute to the sex differences in the immune system of the mitten crab.

Humoral immune responses that mainly occur in hemolymph include proPO system, clotting cascade and a wide array of antimicrobial peptides [68]. The identified female-biased humoral immune factors indicate females have greater lysozyme and PO activities in hemocytes, which is in agreement with those studies in insects [64–66]. PRRs, as a set of germline-encoded receptors, can interact with pathogen associated molecular pattern (PAMP) and activate innate immune response [69]. The higher transcripts of PRRs in males might suggest male mitten crabs could trigger quick and effective defense responses in the presence of pathogens infection. Sex differences in immunity appear to be related to differential reproductive strategies and the resulting resource trade-offs in life history. The identified immune DEGs provide a framework for future research to unravel the mechanism of six-biased immune regulation in crab.

Among the reproduction-related DGEs, two special genes *vitellogenin* (*Vg*) and *insulin-like androgenic gland factor* (*IAG*) were identified. Vg, usually considered as a female specific protein, could serve as energy resource for embryonic development [70]. Apart from its nutritional function, Vg has been shown to play important roles in innate immunity by acting as a multivalent pattern recognition receptor, a bactericidal molecule or an acute phase protein [71]. The *Vg* gene is normally silent in males, but can be activated by estrogen exposure [72,73]. Interestingly, we detected the expression of *Vg* in the normal physiological conditions of male *E*. *sinensis*, which is consistent with the finding in European honey bee *Apis mellifera* [74,75] but contrary to most studies in crustaceans [76–78]. This male-biased expression suggests that *Vg* might have functions in addition to its roles in oocyte maturation and energy supply for embryogenesis. IAG, a key regulator of male sex differentiation in crustaceans [79], is first discovered from *E*. *sinensis* transcriptomes. *EsIAG* shows remarkable structural similarity and sequence homology with other IAGs, suggesting that it is a member of the insulin/insulin-like growth factor family. By qRT-PCR validation, *EsIAG* is expressed in both sexes with a significantly higher level in males. It suggests that *EsIAG* might be not expressed exclusively in the male AG, which is also reported in *S*. *paramamosain* [79], *C*. *sapidus* [80] and *Fenneropenaeus chinensis* [81].

In conclusion, this is the first whole-body, sex-specific transcriptomes of juvenile *E*. *sinensis* using RNA-seq technology. More than 90 million clean reads were obtained, and some candidate genes in sex determination and differentiation were found. A large number of differentially expressed genes between the sexes were identified, and most of them had no obvious sexual function. Many potential SNPs and SSRs were detected that could be used for further gender identification and genetic breeding studies. This study will not only provide valuable genetic resources for the understanding of sexual dimorphism in *E*. *sinensis*, but also facilitate further investigations of functional genomics for this species and other closely related species.

## Supporting Information

S1 Fig Gene Ontology (GO) annotation of all unigenes from *Eriocheir sinensis* transcriptomes. (TIF)

S2 Fig eggNOG functional category of all unigenes from female and male *Eriocheir sinensis* transcriptomes. (TIF)

S3 Fig Expression levels of the identified sex determination genes in female and male *Eriocheir sinensis* transcriptomes. (TIF)

S4 Fig Volcano plot of differentially expressed genes (DEGs) from female and male *Eriocheir sinensis* transcriptomes. For each unigene, the ratio of expression levels (Female vs. Male) is plotted against the-log error rate. The horizontal line indicates the significance threshold (FDR adjusted < 0.05), and the vertical lines indicate the two fold change threshold. Non-differentially expressed genes are shown with orange dots, and DEGs are shown with blue dots.
(TIF)

S5 Fig KEGG categories of differentially expressed genes (DEGs) from female and male *Eriocheir sinensis* transcriptomes. The horizontal line indicates the significance threshold (*P* < 0.05).
(TIF)

S6 Fig Expression pattern of genes involved in sphingolipid metabolism pathway. The up-regulated in male are labeled by green, and the purple color represents genes with no expression differences between female and male *Eriocheir sinensis* transcriptomes.
(TIF)

S7 Fig Amino acid sequence alignment (A) and bootstrapped neighbor-joining (NJ) tree of *Eriocheir sinensis* IAG (EsIAG) with other crustacean IAGs. B and A chains are marked in yellow and green boxes, respectively. The six conserved cysteine residues are highlighted with dark red background and the predicted disulfide bridges are drawn. The species and the GenBank accession numbers are as follow: *Callinectes sapidus* IAG1(AEI72263), *C*. *sapidus* IAG2 (AHM93481), *Cherax destructor* (ACD91988), *Cherax quadricarinatus* (ABH07705), *Fenneropenaeus chinensis* IAG1(AFU60548), *F*. *chinensis* IAG2 (AFU60549), *Jasus edwardsii* (AIM55892), *Litopenaeus vannamei* (AIR09497), Macrobrachium lar (BAJ78349), *Macrobrachium nipponense* IAG1 (AGB56976), *Macrobrachium nipponense* IAG2 (AHA33389), *Macrobrachium rosenbergii* (ACJ38227), *Macrobrachium vollenhovenii* (AHZ34725), *Marsupenaeus japonicus* (BAK20460), *Palaemon pacificus* (BAJ84109), *Palaemon paucidens* (BAJ84108), *Penaeus monodon* (ADA67878), *Portunus pelagicus* (ADK46885), *Sagmariasus verreauxi* (AHY99679) and *Scylla paramamosain* (AIF30295). Three isopods *Armadillidium vulgare* (BAA86893), *Porcellio dilatatus* (BAC57013), *Porcellio scabar* (BAC57012) served as outgroups.
(TIF)

S1 Table Primer sequences and product size of target and reference genes used for real-time PCR. (DOC)

S2 Table Summary statistics of sequencing, assembly and annotation of female and male *Eriocheir sinensis* transcriptomes. (DOC)

S3 Table Summary of simple sequence repeat (SSR) types in female and male *Eriocheir sinensis* unigenes. (DOC)

S4 Table Primary sex-related genes detected in female and male *Eriocheir sinensis* transcriptomes. (XLS)

S5 Table List of SNPs in sex-related genes in female and male *Eriocheir sinensis* transcriptomes. (DOC)

S6 Table List of SSRs in sex-related genes in female and male *Eriocheir sinensis* transcriptomes. (DOC)

S7 Table Differentially expressed genes from female and male *Eriocheir sinensis* transcriptomes. (XLS)
