# Supplementary figures and images for: Comparative Transcriptome Analysis Reveals Sex-Biased Gene Expression in Juvenile Chinese Mitten Crab Eriocheir sinensis
Source: PLoS One. 2015 Jul 20;10(7):e0133068. doi: 10.1371/journal.pone.0133068 (PMC4507985; doi:10.1371/journal.pone.0133068)

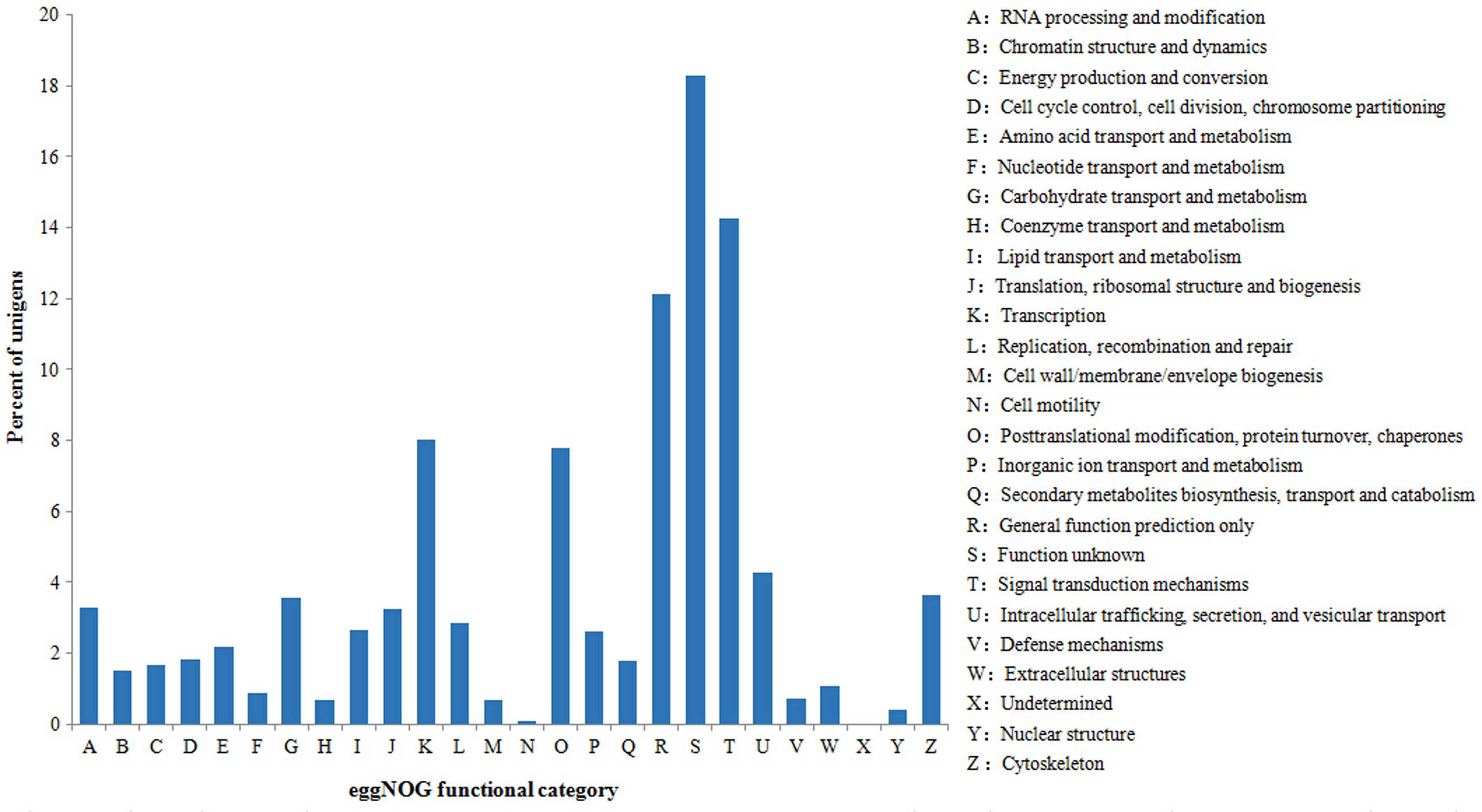

Supplement: S1 Fig — (TIF) [file pone.0133068.s001.tif]

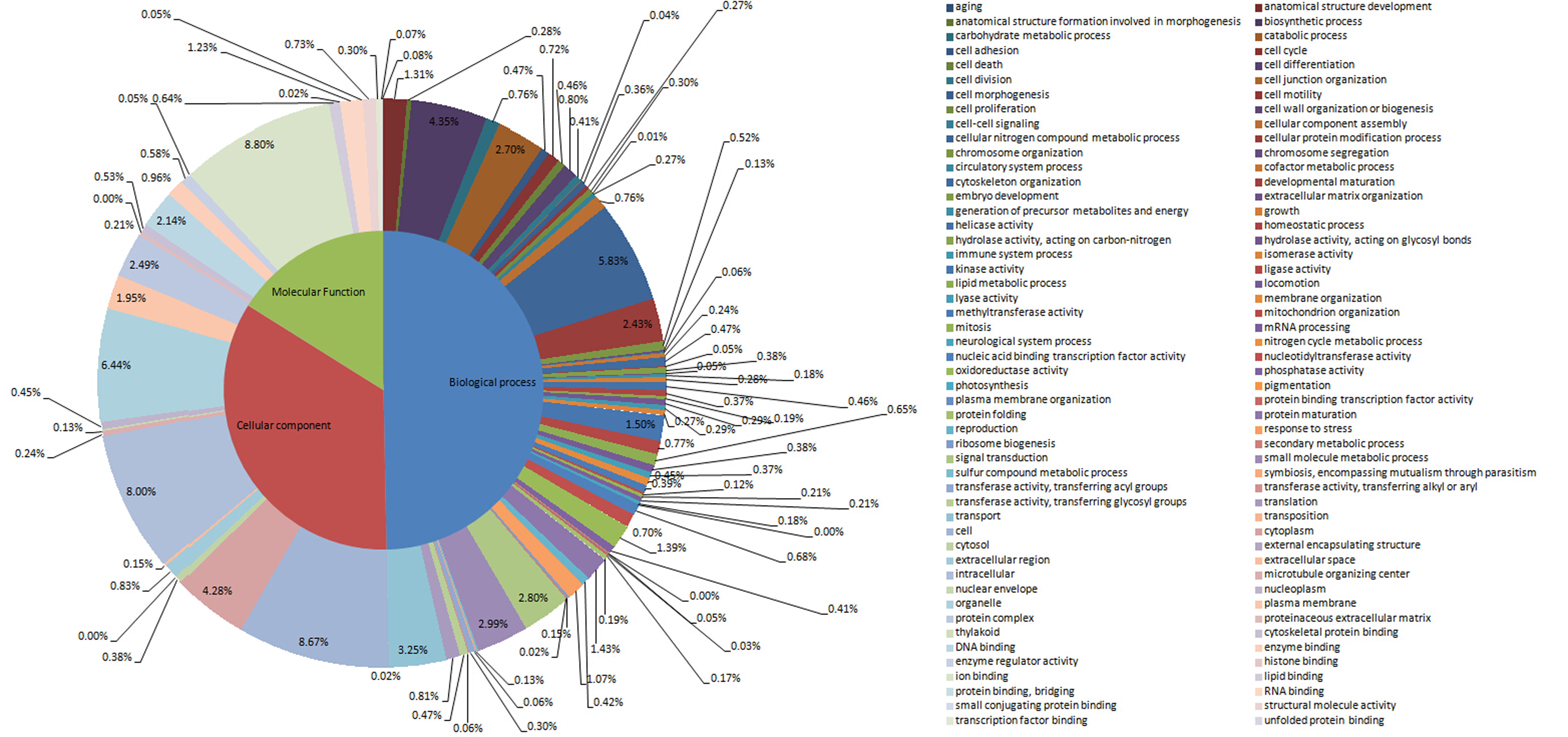

Supplement: S2 Fig — (TIF) [file pone.0133068.s002.tif]

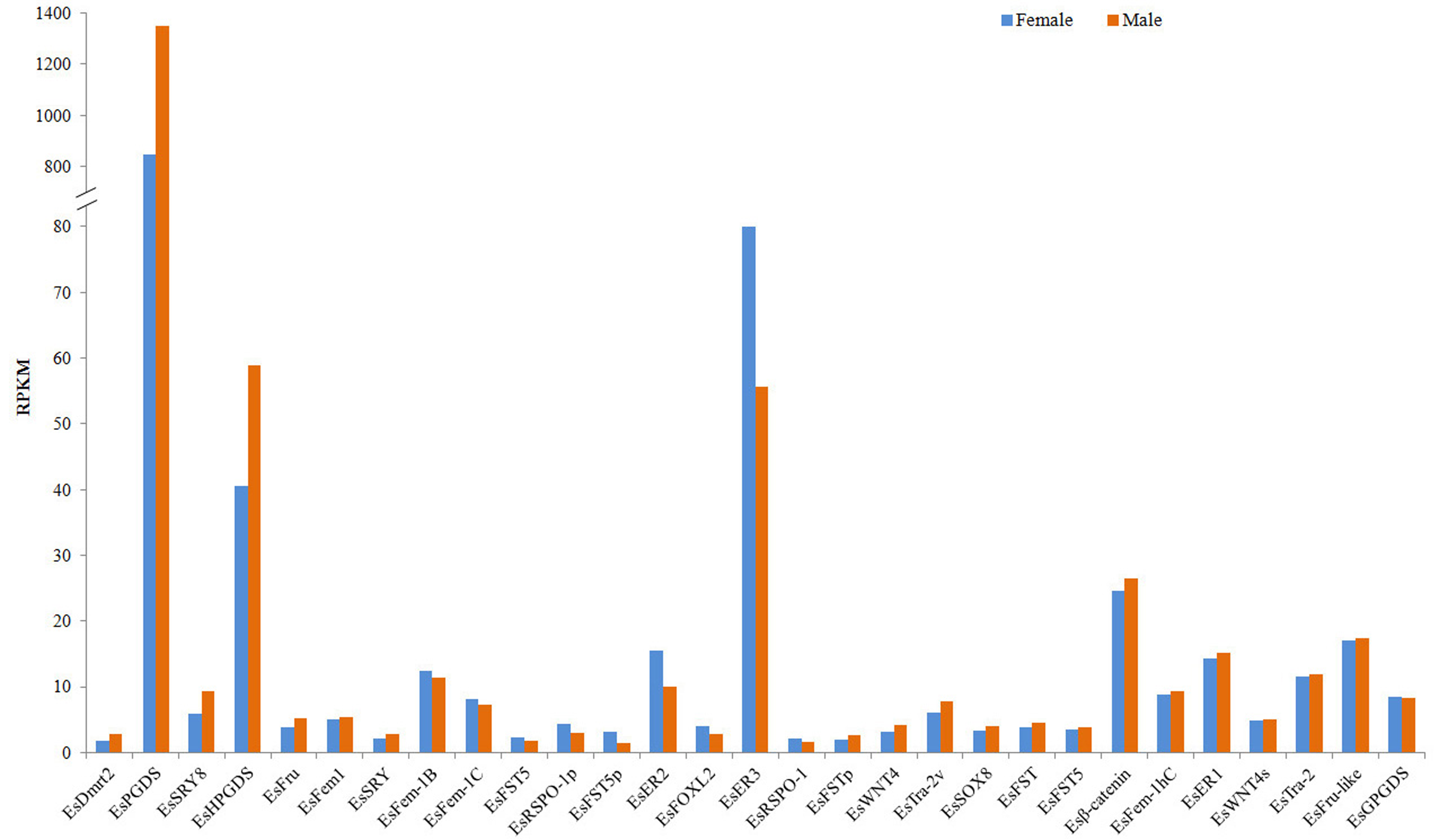

Supplement: S3 Fig — (TIF) [file pone.0133068.s003.tif]

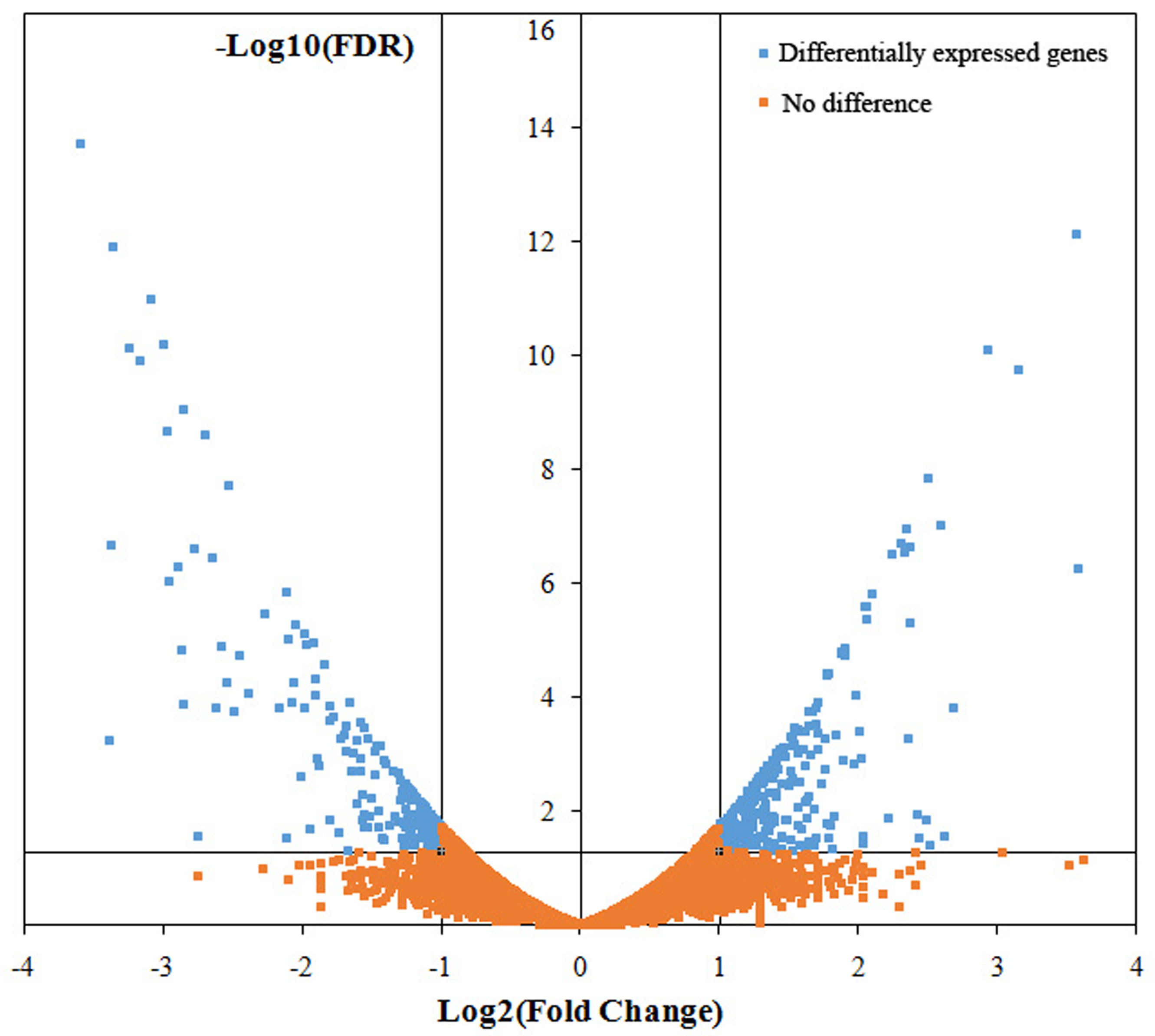

Supplement: S4 Fig — For each unigene, the ratio of expression levels (Female vs. Male) is plotted against the-log error rate. The horizontal line indicates the significance threshold (FDR adjusted < 0.05), and the vertical lines indicate the two fold change threshold. Non-differentially expressed genes are shown with orange dots, and DEGs are shown with blue dots. (TIF) [file pone.0133068.s004.tif]

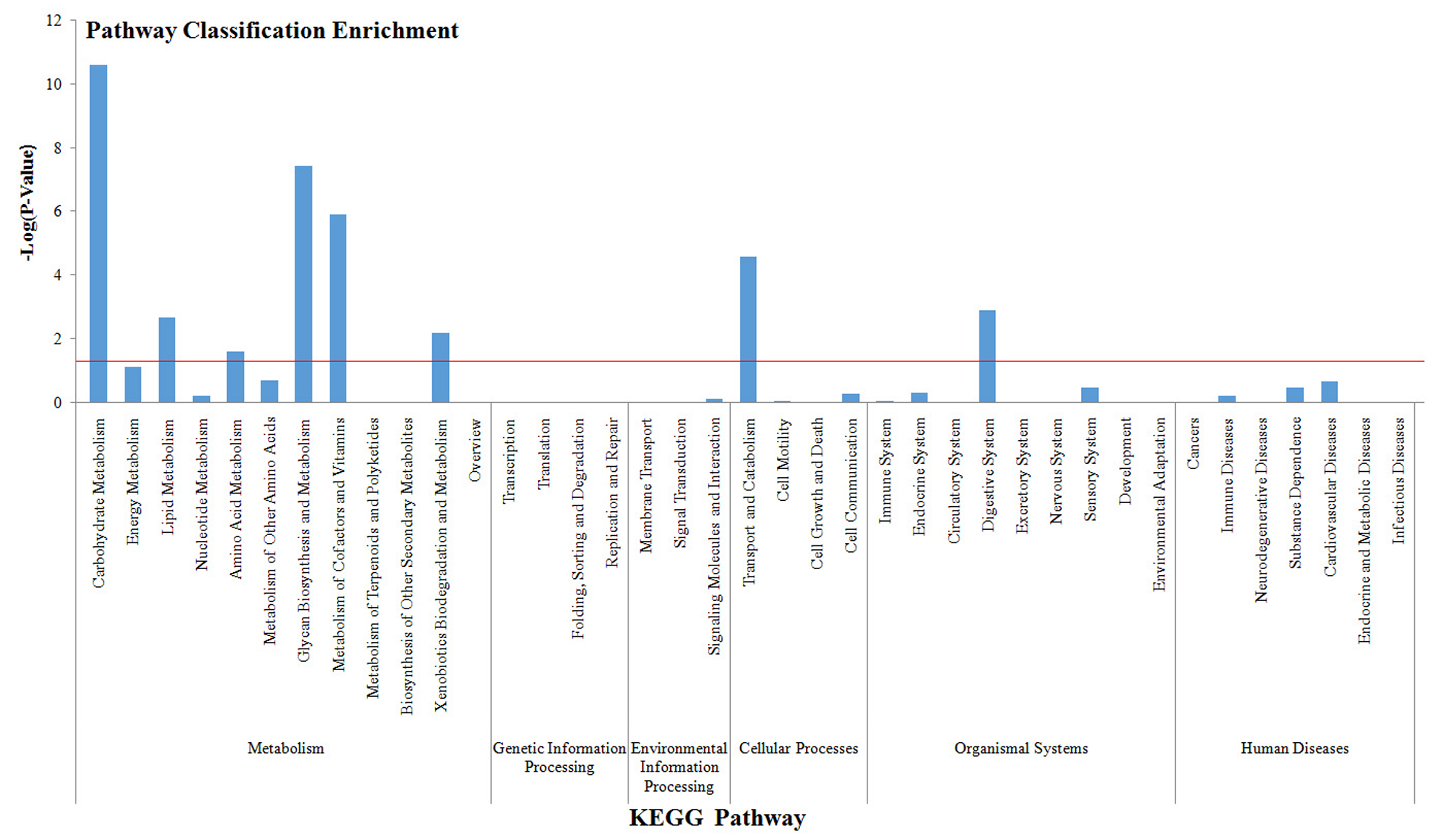

Supplement: S5 Fig — The horizontal line indicates the significance threshold (P < 0.05). (TIF) [file pone.0133068.s005.tif]

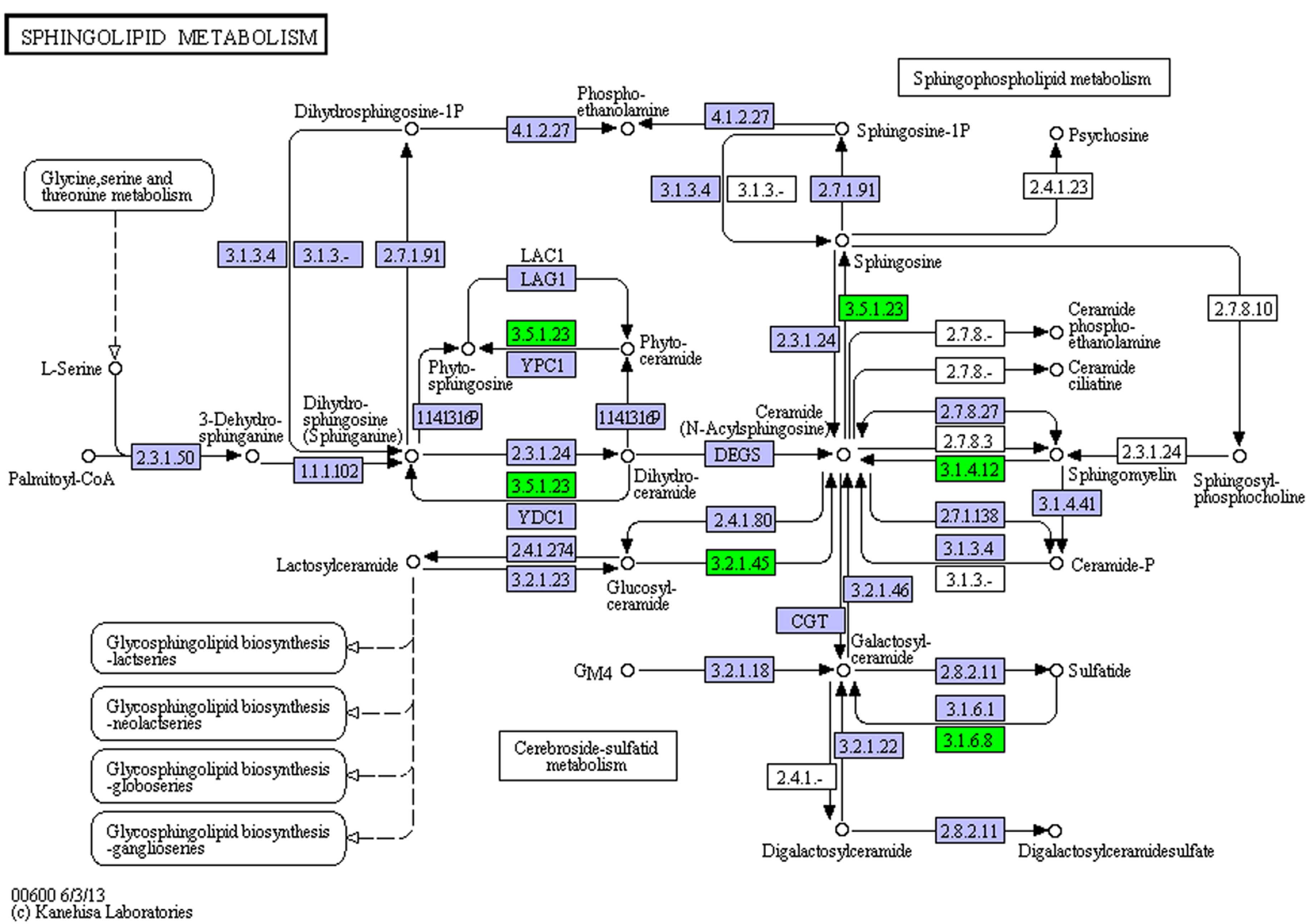

Supplement: S6 Fig — The up-regulated in male are labeled by green, and the purple color represents genes with no expression differences between female and male Eriocheir sinensis transcriptomes. (TIF) [file pone.0133068.s006.tif]

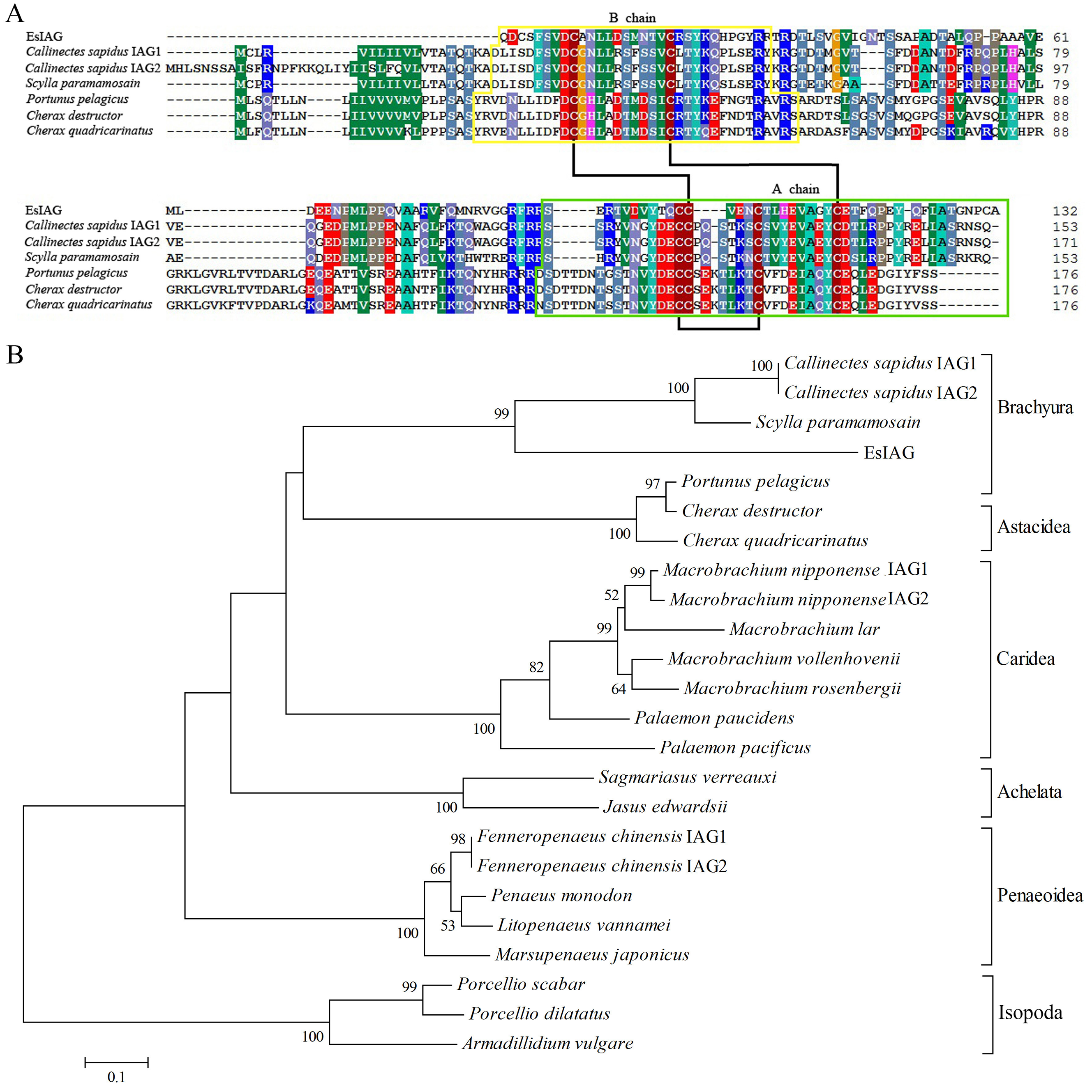

Supplement: S7 Fig — B and A chains are marked in yellow and green boxes, respectively. The six conserved cysteine residues are highlighted with dark red background and the predicted disulfide bridges are drawn. The species and the GenBank accession numbers are as follow: Callinectes sapidus IAG1(AEI72263), C. sapidus IAG2 (AHM93481), Cherax destructor (ACD91988), Cherax quadricarinatus (ABH07705), Fenneropenaeus chinensis IAG1(AFU60548), F. chinensis IAG2 (AFU60549), Jasus edwardsii (AIM55892), Litopenaeus vannamei (AIR09497), Macrobrachium lar (BAJ78349), Macrobrachium nipponense IAG1 (AGB56976), Macrobrachium nipponense IAG2 (AHA33389), Macrobrachium rosenbergii (ACJ38227), Macrobrachium vollenhovenii (AHZ34725), Marsupenaeus japonicus (BAK20460), Palaemon pacificus (BAJ84109), Palaemon paucidens (BAJ84108), Penaeus monodon (ADA67878), Portunus pelagicus (ADK46885), Sagmariasus verreauxi (AHY99679) and Scylla paramamosain (AIF30295). Three isopods Armadillidium vulgare (BAA86893), Porcellio dilatatus (BAC57013), Porcellio scabar (BAC57012) served as outgroups. (TIF) [file pone.0133068.s007.tif]
