# Supplementary material for: Comparative Transcriptome Analysis Reveals Sex-Biased Gene Expression in Juvenile Chinese Mitten Crab Eriocheir sinensis
Source: PLoS One. 2015 Jul 20;10(7):e0133068. doi: 10.1371/journal.pone.0133068 (PMC4507985; doi:10.1371/journal.pone.0133068)
Supplement: S1 Table — (DOC) [file pone.0133068.s008.doc]

**S1 Table. Primer sequences and product size of target and reference genes used for real-time PCR.**

| Gene | Unigene | Forward primer (5′ to 3′) | Reverse primer (5′ to 3′) | Product size (bp) |
| --- | --- | --- | --- | --- |
| *AspRS* | comp59739_c0_seq1 | CAGACGGAATACAGTCAAGC | GGACCATCACATCACCCATA | 148 |
| *NIT2* | comp56883_c0_seq1 | CCACCACAAACACGCCAAACT | CGAGGAGAAGACCGAGAACGT | 204 |
| *Lyz* | comp67931_c0_seq2 | CTCAGTGTCCCGGATGATCTT | AACACCGCTGCCATTAACCAC | 191 |
| *Crustin* | comp54631_c0_seq1 | TGCAAAGACCACAACGACCAA | CCGAAGTCACAAACTCCATCA | 167 |
| *PPAF* | comp72920_c0_seq1 | TAGCATCCTCTCTGTGATTCC | GCTGATGACTCCAAGACCTAT | 158 |
| *Vg* | comp10184_c0_seq1 | TTGGGCAGGCAGTCGTTCTTG | GCCCACGACTTGTACTCAGTT | 258 |
| *ARSA* | comp74420_c0_seq3 | CACGCTCTTGCCGATACAAAC | AACGCCACCGCCTTCATCAAT | 265 |
| *Se-Gpx* | comp66076_c0_seq2 | TGGATGGTAGCGGGTGTATGG | TCCTAAAGAGCGGTTGTGAGT | 142 |
| *CYP3A4* | comp59688_c0_seq1 | CTCACCACTTCATGCCCGAG | GGCTGGGCTAGGGATGACAT | 176 |
| *CLEC* | comp62386_c0_seq1 | AGGAACCGACCTATCTGTGGAAG | AGTAGAAGTCTGGCGGGAAGAG | 152 |
| *LGBP* | comp56987_c0_seq1 | TTCGAGACTCCTTAGCCTGG | AGCGGAGAACTCGACCTGTG | 137 |
| *IAG* | comp61312_c0_seq3 | CAGATGAATCGGGTTGGG | TGGAAGGTTTCGCAGTAG | 119 |
| *β-actin* | Reference gene | GCATCCACGAGACCACTTACA | CTCCTGCTTGCTGATCCACATC | 266 |
