# Supplementary material for: Comparative Transcriptome Analysis Reveals Sex-Biased Gene Expression in Juvenile Chinese Mitten Crab Eriocheir sinensis
Source: PLoS One. 2015 Jul 20;10(7):e0133068. doi: 10.1371/journal.pone.0133068 (PMC4507985; doi:10.1371/journal.pone.0133068)
Supplement: S2 Table — (DOC) [file pone.0133068.s009.doc]

**S2 Table. Summary statistics of sequencing, assembly and annotation of female and male *Eriocheir sinensis* transcriptomes.**

|  | Number | |
| --- | --- | --- |
| **Reads** | Female | Male |
| Total number | 42,979,050 | 47,560,370 |
| Total length (bp) | 4,801,268,098 | 5,278,284,809 |
| Average reads length (bp) | 112 | 111 |
| **Assembly** | Female & Male | |
| Number of contigs | 282,954 | |
| Total length of contigs (bp) | 77,502,939 | |
| Average length of contigs (bp) | 274 | |
| N50 of contigs (bp) | 353 | |
| Number of transcripts | 151,128 | |
| Total length of transcripts (bp) | 92,838,386 | |
| Average length of transcripts (bp) | 614 | |
| N50 of transcripts (bp) | 842 | |
| **Annotation** | Female & Male | |
| Annotated unigenes | 23,349 | |
| Total length of unigenes (bp) | 23,074,969 | |
| Average length of unigenes (bp) | 988 | |
| N50 of unigenes (bp) | 1,375 | |
| Annotation with NR | 23,349 | |
| Annotation with GO | 17,388 | |
| Annotation with eggNOG | 22,101 | |
| Annotation with KO | 9,605 | |
| Annotation with EC | 3,743 | |

NR: non-redundant protein sequences in NCBI; GO: Gene Onotology; eggNOG: evolutionary genealogy of genes: Non-supervised Orthologous Groups; KO: KEGG (Kyoto Encyclopedia of Genes and Genomes) Orthology; EC: Enzyme Commission number.
