# Supplementary material for: Comparative Transcriptome Analysis Reveals Sex-Biased Gene Expression in Juvenile Chinese Mitten Crab Eriocheir sinensis
Source: PLoS One. 2015 Jul 20;10(7):e0133068. doi: 10.1371/journal.pone.0133068 (PMC4507985; doi:10.1371/journal.pone.0133068)
Supplement: S3 Table — (DOC) [file pone.0133068.s010.doc]

**S3 Table. Summary of simple sequence repeat (SSR) types in female and male *Eriocheir sinensis*** unigenes.

| SSR type | Number | Percentage (%) | Major motif | Number | Percentage (%) |
| --- | --- | --- | --- | --- | --- |
| **Perfect** |  |  |  |  |  |
| Dinucleotides | 1561 | 37.3 | AC/GT | 1048 | 67.1 |
| Trinucleotides | 2286 | 54.6 | AGG/CCT | 718 | 31.4 |
| Tetranucleotides | 81 | 1.9 | ACAT/ATGT | 16 | 19.8 |
| **Compound** | 262 | 6.3 |  |  |  |
| **Total** | 4190 |  |  |  |  |
