# Supplementary material for: Comparative Transcriptome Analysis Reveals Sex-Biased Gene Expression in Juvenile Chinese Mitten Crab Eriocheir sinensis
Source: PLoS One. 2015 Jul 20;10(7):e0133068. doi: 10.1371/journal.pone.0133068 (PMC4507985; doi:10.1371/journal.pone.0133068)
Supplement: S5 Table — (DOC) [file pone.0133068.s012.doc]

**S5 Table. List of SNPs in sex-related genes in female and male *Eriocheir sinensis*** transcriptomes.

| Sex | Unigene | Gene description | Position | Allele variations | Frequencies |
| --- | --- | --- | --- | --- | --- |
| Female | comp21432_c0_seq1 | SRY-box containing gene 8 | 145 | C/T | 72.0/28.0 |
|  | comp26865_c0_seq1 | RSPO-1 | 74 | C/T | 55.6/44.4 |
|  | comp58110_c0_seq1 | Vitelline membrane outer layer protein I | 393 | C/T | 99.9/0.1 |
|  | comp58110_c0_seq1 |  | 668 | T/C | 99.9/0.1 |
|  | comp59942_c0_seq1 | Testis-expressed sequence 2 protein | 363 | A/T | 68.8/31.2 |
|  | comp60878_c0_seq2 | Spermatogenesis-associated protein 5 | 325 | G/A | 60.0/40.0 |
|  | comp60878_c0_seq2 |  | 338 | G/T | 58.3/41.7 |
|  | comp61811_c0_seq1 | Testis-specificprotein A14 protein | 375 | G/T | 52.9/47.1 |
|  | comp61959_c0_seq1 | Transcription factor SOX-14 | 96 | T/A | 87.5/12.5 |
|  | comp61959_c0_seq1 |  | 312 | G/C | 86.5/13.5 |
|  | comp62786_c0_seq1 | Transcription factor SOX-5 | 282 | A/T | 69.2/30.8 |
|  | comp64396_c0_seq1 | Spermatogenesis-associated protein 5 | 154 | A/G | 61.5/38.5 |
|  | comp64833_c0_seq3 | DnaJ chaperonine | 1366 | G/A | 79.3/20.7 |
|  | comp66701_c0_seq3 | Testis derived transcript (3 LIM domains) | 1221 | G/C | 68.8/31.2 |
|  | comp67693_c0_seq1 | Nuclear autoantigenic sperm protein | 778 | C/G | 88.9/11.1 |
|  | comp68590_c0_seq2 | Round spermatid basic protein 1 | 557 | C/G | 99.9/0.1 |
|  | comp69264_c0_seq3 | Zinc finger protein 76 (expressed in testis) | 368 | C/T | 76.5/23.5 |
|  | comp69550_c0_seq1 | Spermatogenesis-associated protein 20 | 493 | C/A | 72.7/27.3 |
| Male | comp18195_c0_seq1 | Dmrt2 | 126 | T/C | 90.9/9.1 |
|  | comp33591_c0_seq2 | Virilizer | 169 | C/T | 60.0/40.0 |
|  | comp56545_c0_seq1 | FOXL2 | 1054 | C/A | 66.7/33.3 |
|  | comp61312_c0_seq3 | Insulin-like androgenic gland factor IAG | 290 | C/G | 53.9/46.1 |
|  | comp61325_c0_seq1 | Male-specific lethal 1-like 1 MSL1 | 1360 | G/A | 57.1/42.9 |
|  | comp62221_c0_seq1 | Hsp40 | 1043 | A/G | 99.9/0.1 |
|  | comp67300_c0_seq1 | Motile sperm domain-containing protein 2 | 782 | G/T | 54.2/45.8 |
|  | comp67327_c0_seq1 | Androgen-induced gene 1 protein | 829 | A/T | 72.2/27.8 |
|  | comp68590_c0_seq2 | Round spermatid basic protein 1 | 728 | A/G | 64.3/35.7 |
|  | comp68926_c0_seq1 | Oocyte zinc finger protein XlCOF7.1 | 513 | A/G | 92.9/7.1 |
|  | comp68926_c0_seq1 |  | 788 | C/G | 63.2/36.8 |
|  | comp69264_c0_seq3 | Zinc finger protein 76 (expressed in testis) | 431 | G/T | 77.8/22.2 |
|  | comp69550_c0_seq3 | Spermatogenesis-associated protein 20 | 160 | C/A | 58.3/41.7 |
|  | comp69550_c0_seq3 |  | 980 | T/C | 52.6/47.4 |
|  | comp69550_c0_seq3 |  | 1406 | A/G | 82.4/17.6 |
|  | comp69550_c0_seq3 |  | 1421 | A/G | 92.9/7.1 |
|  | comp69685_c0_seq1 | Male reproductive-related microfibril-associated protein | 279 | G/C | 85.2/14.8 |
|  | comp69685_c0_seq1 |  | 562 | T/C | 81.3/18.7 |
|  | comp70181_c0_seq3 | Male sterility domain-containing protein | 1499 | G/A | 92.9/7.1 |
|  | comp70417_c0_seq1 | DEAD box ATP-dependent RNA helicase | 2220 | A/G | 63.2/36.8 |
|  | comp71568_c1_seq2 | Androgen-induced gene 1 protein-like isoform 2 | 1014 | C/A | 99.9/0.1 |
|  | comp71636_c0_seq3 | SRY interacting protein 1 | 867 | G/A | 90.9/9.1 |
|  | comp72150_c0_seq7 | Histone H2B, gonadal-like | 287 | A/G | 63.6/36.4 |
