# Supplementary material for: Comparative Transcriptome Analysis Reveals Sex-Biased Gene Expression in Juvenile Chinese Mitten Crab Eriocheir sinensis
Source: PLoS One. 2015 Jul 20;10(7):e0133068. doi: 10.1371/journal.pone.0133068 (PMC4507985; doi:10.1371/journal.pone.0133068)
Supplement: S6 Table — (DOC) [file pone.0133068.s013.doc]

**S6 Table. List of SSRs in sex-related genes in female and male *Eriocheir sinensis*** transcriptomes.

| Unigene | Gene description | SSR type | SSR | size |
| --- | --- | --- | --- | --- |
| comp21432_c0_seq1 | SRY-box containing gene 8 | c | (GTG)5agggtgctggtggtggatcaggcctggcggaggtggtacagcgtggagaggtttgggtggcagtggagaaaagacttgatatttct(TCC)6 | 119 |
| comp53351_c0_seq1 | Glutathione-dependent prostaglandin D synthase | p3 | (CCT)5 | 15 |
| comp53351_c0_seq1 |  | p2 | (CA)10 | 20 |
| comp55365_c1_seq1 | Estradiol receptor-like protein 2 | p3 | (GTT)7 | 21 |
| comp58387_c0_seq1 | Prostaglandin D synthase | c | (CCT)7(TCT)5 | 36 |
| comp62044_c0_seq1 | Male sterility domain-containing protein | p3 | (GGA)7 | 21 |
| comp62285_c0_seq3 | Sperm-associated antigen 7 | p3 | (ACC)5 | 15 |
| comp64396_c0_seq2 | Spermatogenesis-associated protein 5 | p3 | (CCT)7 | 21 |
| comp67693_c0_seq1 | Nuclear autoantigenic sperm protein | c | (GAG)5ggaaaggaggaagctgccgatggtaaaaagaaggcgaagaaggaagaaggagctgaggaaggagagggcgagaacaca(GAG)5 | 108 |
| comp67693_c0_seq2 | Nuclear autoantigenic sperm protein | p3 | (GAG)5 | 15 |
| comp69264_c0_seq3 | Zinc finger protein 76 (expressed in testis) | p2 | (TG)6 | 12 |
| comp70417_c0_seq1 | DEAD box ATP-dependent RNA helicase | p3 | (CTT)5 | 15 |
| comp71636_c0_seq3 | SRY interacting protein 1 | p3 | (CCA)7 | 21 |
| comp71636_c0_seq3 |  | p3 | (GCC)5 | 15 |
| comp72717_c0_seq1 | male-specific transcription factor FRU-MB | p3 | (GAG)5 | 15 |
| comp73043_c0_seq2 | male-specific lethal 3-like 2 | p3 | (GGA)5 | 15 |
| comp73297_c0_seq1 | Testis-expressed sequence 10 protein | p2 | (AC)10 | 20 |
| comp73970_c3_seq4 | Spermatogonial stem-cell renewal factor | c | (TTC)6tt(TTC)7 | 41 |
| comp73984_c0_seq5 | Hematopoietic prostaglandin D synthase | c | (CTA)5ctgctgctatcacggactcctgctatcgactacttataattttgtgttcaaagattttactgcatggcttttctgcttgttcgtgta(TGTC)5tatgtctgtctatatata(T)10ccttcgctatctaaaacacaatattcagaagaacgcgcttggaatcggaaccgacggacaaacaccgatttagcgga(GGT)5 | 242 |
| comp74843_c0_seq1 | Mago nashi 2 | p2 | (CA)10 | 20 |
| comp74865_c0_seq3 | DEAD box ATP-dependent RNA helicase | p2 | (TG)8 | 16 |
| comp75937_c2_seq2 | Dosage compensation regulator isoform 2 | p3 | (TCC)7 | 21 |
| comp8990_c0_seq1 | Spermatogenesis-associated protein 20 | p2 | (CT)6 | 12 |
